# Systematic Review of the Therapeutic Role of Apoptotic Inhibitors in Neurodegeneration and Their Potential Use in Schizophrenia

**DOI:** 10.3390/antiox11112275

**Published:** 2022-11-17

**Authors:** Constanza Morén, Nina Treder, Albert Martínez-Pinteño, Natàlia Rodríguez, Néstor Arbelo, Santiago Madero, Marta Gómez, Sergi Mas, Patricia Gassó, Eduard Parellada

**Affiliations:** 1 Barcelona Clínic Schizophrenia Unit (BCSU), Institute of Neuroscience, Psychiatry and Psychology Service, Hospital Clínic of Barcelona, University of Barcelona, 08036 Barcelona, Spain; 2 Clinical and Experimental Neuroscience Area, The August Pi i Sunyer Biomedical Research Institute (IDIBAPS), 08036 Barcelona, Spain; 3 U722 Group, Centro de Investigación Biomédica en Red de Enfermedades Raras, CIBERER, Carlos III Health Institute, 28029 Madrid, Spain; 4 Department of Basic Clinical Practice, Pharmacology Unit, University of Barcelona, 08036 Barcelona, Spain; 5 Faculty of Psychology and Neuroscience, Maastricht University, 6211 LK Maastricht, The Netherlands; 6 G04 Group, Centro de Investigación Biomédica en Red de Salud Mental (CIBERSAM), Carlos III Health Institute, 28029 Madrid, Spain; 7 Department of Psychiatry, Servizo Galego de Saúde (SERGAS), 36001 Pontevedra, Spain

**Keywords:** apoptosis, apoptotic inhibitors, schizophrenia, systematic review, therapy

## Abstract

Schizophrenia (SZ) is a deleterious brain disorder affecting cognition, emotion and reality perception. The most widely accepted neurochemical-hypothesis is the imbalance of neurotransmitter-systems. Depleted GABAergic-inhibitory function might produce a regionally-located dopaminergic and glutamatergic-storm in the brain. The dopaminergic-release may underlie the positive psychotic-symptoms while the glutamatergic-release could prompt the primary negative symptoms/cognitive deficits. This may occur due to excessive synaptic-pruning during the neurodevelopmental stages of adolescence/early adulthood. Thus, although SZ is not a neurodegenerative disease, it has been suggested that exaggerated dendritic-apoptosis could explain the limited neuroprogression around its onset. This apoptotic nature of SZ highlights the potential therapeutic action of anti-apoptotic drugs, especially at prodromal stages. If dysregulation of apoptotic mechanisms underlies the molecular basis of SZ, then anti-apoptotic molecules could be a prodromal therapeutic option to halt or prevent SZ. In fact, risk alleles related in apoptotic genes have been recently associated to SZ and shared molecular apoptotic changes are common in the main neurodegenerative disorders and SZ. PRISMA-guidelines were considered. Anti-apoptotic drugs are commonly applied in classic neurodegenerative disorders with promising results. Despite both the apoptotic-hallmarks of SZ and the widespread use of anti-apoptotic targets in neurodegeneration, there is a strikingly scarce number of studies investigating anti-apoptotic approaches in SZ. We analyzed the anti-apoptotic approaches conducted in neurodegeneration and the potential applications of such anti-apoptotic therapies as a promising novel therapeutic strategy, especially during early stages.

## 1. Introduction

Schizophrenia (SZ) is a heterogeneous psychiatric disorder with unclear etiology affecting ~1% of the population worldwide [1]. The few pharmacological treatments available mainly target the positive psychotic symptoms, but not the negative and cognitive symptoms. To gain a complete understanding of SZ, the integration of multidisciplinary approaches through molecular biology, genetics, epigenetics, environmental factors, neuroimaging, cell and animal models, translational clinical and epidemiological research is needed. According to the neurodevelopmental hypothesis of SZ [2], early processes such as abnormal neurogenesis, neuronal migration, dendritic arborization or axonal outgrowth would affect the formation of the neural circuits. During adolescence and young adulthood, postnatal brain maturation abnormalities, including excessive dendritic spine pruning [3], could eventually account for the onset of SZ symptoms [4]. In order to study dendritic pruning during brain maturation, several key neurodevelopmental animal models of SZ have been developed. Some neurodevelopmental models of SZ include the maternal immune activation model [5] or the use of antagonists of the n-methyl-D-aspartate (NMDA) receptors, such as MK-801 [6] ketamine [7] and phencyclidine [8,9] or prenatal methylazoxymethanol acetate exposure [10,11,12,13], with the latter being one of the most validated neurodevelopmental model of SZ, in terms of face, construct and predictive validity [14,15,16,17,18].

### 1.1. Synaptic Pruning in SZ

**Figure 1:**
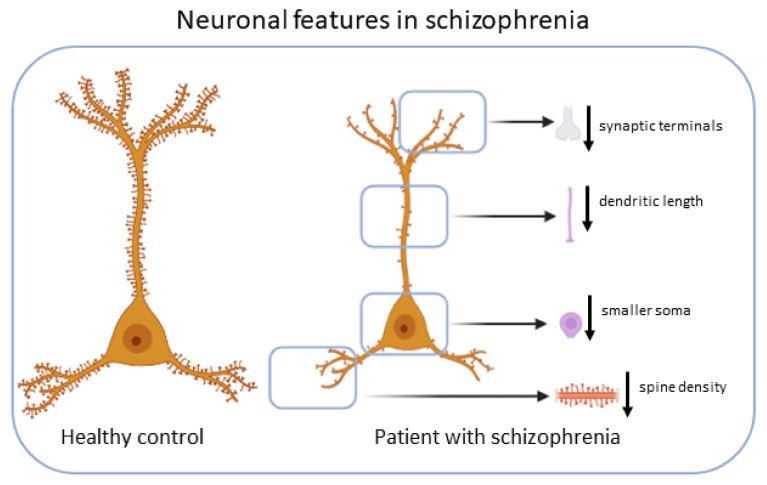
Neuronal features in SZ. Cortical pyramidal neurons from subjects with schizophrenia exhibit smaller soma volume, decreased spine density, decreased dendritic length and decreased terminals.

Cortical pyramidal neurons from subjects with SZ exhibit different anatomical features [19] (Figure 1).

The hypotheses of the underlying etiopathogenesis of SZ have been repeatedly reformulated and yet continue to be revisited. We recently reviewed a reassessment of the synaptic over-pruning hypothesis of SZ [20]. The glutamate hypothesis, accounting for the mostly prodromal negative symptoms, arose more recently as compatible with the initial hypothesis of a dopamine storm explaining the positive symptoms. Glutamate acts as the main neurotransmission modulator via NMDA/α-amino-3-hydroxy-5-methyl-4- isoxazolepropionic acid (AMPA) receptors under physiological conditions, but may trigger loss of integrity of dendritic spines upon dysregulation. During a critical neurodevelopmental period, there is dysregulation between excitatory glutamatergic pyramidal neurons and inhibitory gamma-aminobutyric acid (GABAergic) interneurons, leading to excessive local glutamate release [20]. Glutamate dysregulations, via its NMDA/AMPA receptors, negatively impact the integrity of the dendritic spines, with subsequent excessive dendritic pruning with the activation of local apoptosis machinery. Over-pruning of dendritic spines, together with aberrant synaptic plasticity may lead to neural misconnections and suboptimal synaptic function, promoting clinical symptoms [20]. Indeed, *postmortem* studies have reported reduced number of dendritic spines, especially on pyramidal neurons located in layer-III of the prefrontal cortex, in the superior temporal gyrus, and in hippocampal subfields (CA3) [3,21]. Taken together, the driving hypothesis of glutamate storms originating from uncontrolled extrapyramidal neuron firing due to inefficient inhibitory regulation of GABAergic-interneurons highlights the importance of pharmacological interventions during the prodromal phase of disease and apoptotic targeting. Accordingly, the use of anti-apoptotic drugs has been reported to prevent the loss of GABAergic interneurons [22].

**Figure 2:**
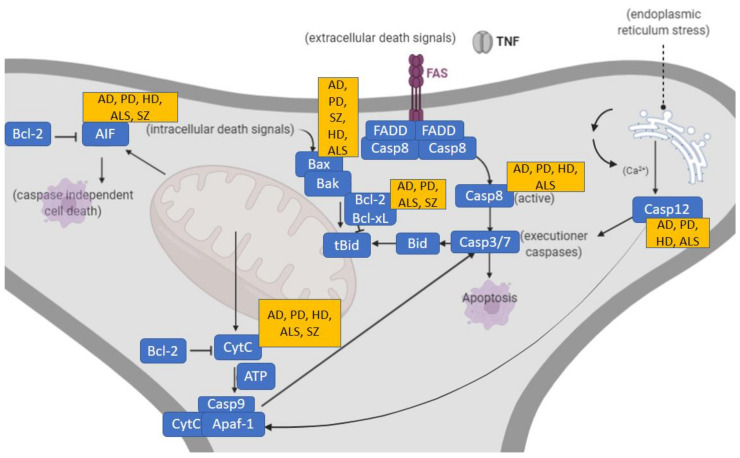
Two prototypical apoptotic pathways (extrinsic and intrinsic) initiated by separate events converge at a common place to execute apoptosis. Main associations of these apoptotic pathways to the neurodegenerative disorders AD, PD, HD as well as SZ are indicated. Death receptor pathways triggered by binding of death receptors by their ligands (tumor necrosis factor (TNF, FasL) that results in receptor clustering and recruitment of adaptor proteins, leading to the activation of initiator caspase-8. The intrinsic pathway responds to a variety of cellular stress signals that act on mitochondria to cause leakage of pro-apoptotic effectors like cytochrome c and apoptosis-inducing factor (AIF). Cytochrome c binds to Apaf-1 in the presence of ATP to form the “apoptosome”, which subsequently recruits and activates initiator pro-caspase-9. AIF translocates to the nucleus to cause high molecular weight DNA fragmentation in a caspase-independent manner. Both pathways activate effector caspases-3 and -7. Endoplasmic reticulum stress causes the activation and release of caspase-12, leading to apoptotic cell death. AIF, apoptosis-inducing factor; AD, Alzheimer’s disease; ALS, amyotrophic lateral sclerosis; Apaf, apoptotic protease activating factor; Bak, Bcl-2 homologous antagonist killer; Bax, Bcl-2 associated X protein; Bcl, B-cell lymphoma; Bid, BH3 interacting domain death agonist; Casp, caspase; CytC, cytochrome c; FADD, Fas-associated-death-domain protein; FAS, cysteine-rich transmembrane protein CD95; HD, Huntington’s disease; PD, Parkinson’s disease; SZ, schizophrenia; tBid, truncated Bid; TNF, tumor necrosis factor.

Dendritic pruning, however, is not restricted to pathologic contexts. Physiologically, dendritic pruning corresponds to a highly regulated homeostatic neurodevelopmental process to either favor or delete certain brain connections underpinning synaptic plasticity during normal growth. Significant cell death occurs during early development of the nervous system with over half of all developing neurons dying via apoptosis [23]. Interestingly, aberrant dendritic apoptosis preceding neuronal death has also been found in the hippocampus and prefrontal cortex in the early stages of neurodegenerative diseases such as Alzheimer’s disease (AD) [24]. Even though the underlying molecular mechanisms are not fully understood [25], synaptic pruning exhibits molecular features in common with apoptosis [26]. It is worth mentioning that microglia and immune responses also contribute to the loss of dendritic spine density in SZ [27], although this is beyond the scope of this review. In any case, during dendritic apoptosis, apoptotic molecules generate the signals that attract microglia for removal of synaptic debris, suggesting that microglial phagocytosis of synapses occurs downstream in this apoptotic process [28]. Such apoptotic processes are often regulated by a complex molecular cascade of cysteine proteases, the so-called caspases (caspase-1–15, of which -2,-8,-9,-10 are initiators and -3-6-7 are effectors) [29]. The two main apoptotic pathways [24], the molecular interactions [30,31] and involvement of inhibitor-of-apoptosis (IAP) molecules [32] are herein represented (Figure 2).

### 1.2. Apoptotic Molecular Studies in Neurodegenerative Disorders and Overlap with SZ

Apoptotic molecular changes and the involvement of apoptotic proteins have been observed in neurodegenerative disorders, including AD [33], Parkinson’s disease (PD) [34], Huntington’s disease (HD) [35] and amyotrophic lateral sclerosis (ALS) [36]. Some or the specific molecular apoptotic changes occurring in these neurodegenerative disorders, also observed in SZ, will be extensively discussed in Section 3 and briefly summarized in Figure 2. Importantly, at a genetic level, different known risk alleles related to apoptosis have been found to be associated with SZ in the recent literature, including apoptotic mediator *BCL11B*, *BCL2L12*, *BNIP3L* and *ENOX1* genes [37]. 

The clear association between neuronal death and neurodegenerative mechanisms has led to the widespread use of anti-apoptotic therapeutic strategies in models of classic neurodegenerative disorders, including AD [38], PD [39], HD [40] and ALS [41]. However, neuronal death is a complex process that is not restricted to a unique type of mechanism, quite the opposite, it may occur via a complex and strikingly high number of molecular events [24]. While multiple molecular pathways lead to the activation of the caspases, the mitochondrial intrinsic pathway is mostly associated with neuronal apoptosis [19,31]. Regulation of mitochondrial outer membrane permeabilization and intrinsic apoptosis in neurons appears to center on control of Bax activation (Table S1). Evidence of a main role of the mitochondrial apoptotic machinery in SZ etiopathogenesis has been reported in the literature [42,43], placing mitochondrial anti-apoptotic molecules as a potential surrogate therapeutic option. Apoptotic activity can be both preceded and triggered by a broad panoply of stimuli including pro-inflammatory cytokines, excitotoxicity (including glutamate excitotoxicity earlier related to SZ), neurotrophin withdrawal, abnormal calcium concentrations and mitochondrial dysfunction with subsequent oxidative stress [44]. In fact, the inhibitory GABAergic-interneurons expressing parvalbumin are highly sensitive to oxidative stress and require a highly regulated antioxidant system to neutralize the overproduction of reactive oxygen species (ROS) generated by mitochondria [45]. Interestingly, all the aforementioned apoptotic triggers are present in SZ, suggesting apoptosis as a key factor of SZ onset.

### 1.3. Apoptotic Molecular Studies in SZ

Despite the underlying mechanisms leading to synaptic dysfunction in SZ being uncertain, evidence suggests that dysregulation of neuronal apoptosis may contribute to its pathophysiology [19,46]. It has been hypothesized that apoptotic activity contributes to the evidence for reduced grey matter volume and synaptic deficits in SZ [19,47]. Additionally, synaptic/dendritic neuronal loss could be explained by increased susceptibility to apoptosis in SZ. The basis for the apoptotic features in SZ is widely supported. First, there is a 50% increase in the Bax/Bcl-2 ratio as an apoptotic indicator (via cytochrome-c release) in the temporal cortex (area 21, middle temporal gyrus) of SZ patients [48]. Second, there is a 30% decrease in Bcl-2 levels in the temporal cortex in SZ [49]. This suggests that cortical neurons and synapses in patients with SZ may have less neuroprotection given that Bcl-2 can exert both neuroprotective and neurotrophic effects [19].

Although caspase activation is often considered a precursor to rapid cell death, the emerging concept of synaptic apoptosis suggests that apoptotic activation can be localized to synapses of distal neurites without inducing immediate neuronal death or involving the neuronal cell body [19,50]. Interestingly, caspase-3 activity has been associated with normal physiological activity, including synaptic plasticity [19]. It is of note that local apoptosis in SZ occurs in the absence of neuronal cell loss and without changes in the number of pyramidal neurons [51]. This is in line with animal models that have shown local caspase activity in neurons in which caspases were confined to the dendritic compartment of pruning instead of the soma or axonal areas [26]. NMDA receptor activation can trigger local dendritic apoptosis [52], and following focal application of glutamate to distal dendrites in vitro, a localized increase in caspase-3 activity was seen without propagation to the neuronal soma [50]. This local activation of apoptosis mediated by caspase-3 within distal dendrites is enough to prune dendritic spines and branches locally [53]. If apoptotic activity contributes to synaptic and neuritic elimination, then there must also be a mechanism to limit the proliferation of the caspase cascade to the rest of the neuron. The induction of endogenous caspase-inhibitors may be present in neuronal cytoplasm [54]. Importantly, the different molecular findings constituting the molecular basis of SZ, including glutamate excitotoxicity, oxidative stress or lack of neurotrophic factors may converge at the apoptotic endpoint [45]. Results from our research group add further evidence supporting the apoptotic hallmarks in the disease. We described increased apoptotic susceptibility in primary fibroblasts from a skin biopsy of naïve patients with a first-psychotic episode [55] and found a correlation between altered apoptotic markers with both the volume of certain brain regions and glutamate/glutamine-neurometabolites [56]. Additionally, we observed alterations in the expression of genes involved in apoptotic pathways [57]. The consistent findings in the literature [58], supported by our group, suggesting that dysregulation of apoptotic mechanisms underlies the molecular basis of SZ, led us to propose anti-apoptotic molecules as a challenging prodromal therapeutic option to halt onset and/or SZ progression.

This systematic review is therefore justified based on: i) the neurodevelopmental changes derived from excessive dendritic pruning via local activation of the apoptosis machinery occurring around the onset of SZ, and ii) the efficacy of apoptotic-inhibitors in classic neurodegenerative disorders, including AD, PD, HD and ALS. We have gathered the scientific data reported in the literature that used anti-apoptotic molecules to halt neurodegeneration and have analyzed those treatments with anti-apoptotic properties against SZ to further elucidate their potential as novel effective therapeutic approaches targeting SZ. 

## 2. Materials and Methods

This systematic review was conducted in accordance with PRISMA guidelines [59]. The 24-step guide for systematic review and meta-analysis in medical research was followed [60], including the definition of research question, team and search strategy, selection criteria, data collection form, study protocol and registration (PROSPERO ID CRD42021238668). The collection of all references and abstracts, searched in multiple databases, was gathered in a single file, and elimination of duplicates was performed. Two reviewers screening title and abstract were used. Collection, comparison and selection for retrieval was developed by the two independent reviewers. The full texts of the references selected based on titles and abstracts were retrieved and the quality of the studies was considered. Articles were identified by searching for titles in Web of Science (WoS), PUBMED and SCOPUS databases by using the following research terms: “schizophrenia” OR “psychosis” OR “neurodegeneration” OR “neurodegenerative disorders” OR “neurodevelopmental disorders” AND “caspase inhibitor” OR “pan-caspase inhibitor” OR “anti-apoptotic molecules” OR “cytochrome c inhibitor” OR “apoptotic inhibitor” OR “mitochondrial apoptotic channel inhibitors”. The search was restricted to English, Spanish or German language journal articles with in vitro, human and/or animal subjects, published between 1990 and 2021. Blinding during data extraction between the review authors was achieved making use of Rayyan software for systematic reviews (Qatar Computing Research Institute, HBKU, Doha, Qatar) [61] which also helps expedite the initial screening of abstracts and titles using a process of semi-automation while incorporating a high level of usability.

The searches returned 917 records after duplicates were removed. Screening summary showed 227 included articles, 647 excluded articles and 43 potential articles for reviewer 1, and 243 included articles and 674 excluded articles, for reviewer 2. After blind was turned off, discrepancies were further solved through additional rigorous assessment criteria. The number of studies included throughout the identification, screening, eligibility and inclusion processes (n = 124) is provided as a flow diagram (Figure S1).

### 2.1. Inclusion and exclusion criteria 

Those studies presenting neurological disorders, with a component of neurodegeneration, using interventions with anti-apoptotic compounds were included, with special focus on molecular findings at the neuronal level. Scoping, systematic and literature reviews were considered for descriptive purposes. Similarly, those studies without quantifiable data to be extracted (n = 54) and in silico studies (n = 2) were considered for descriptive purposes. Ischemic or stroke models were excluded due to a distal etiopathogenic origin with the disease of interest. Studies not controlling for major confounding factors such as drug treatment, drug abuse, and different co-morbidities, or that lacked age or gender-matched controls were not considered. Also, studies that did not allow access to the full text were excluded. 

### 2.2. Primary and Secondary Outcomes 

The main primary outcome parameters of interest for this evaluation were of a biological nature, as measured by quantification of occurrence of local neuronal viability, death, loss of dendritic arborization, spine shrinkage, synaptic pruning and function with inclusion of both continuous and dichotomous data. Since some interventions of this nature have a close relationship to mitochondria, mitochondrial parameters, such as mitochondrial respiratory chain function or oxidative stress parameters and intrinsic apoptosis with involvement of Bax, Bcl-2 and cytochrome c release were considered. Thus, the literature was selected in line with these primary outcomes: Molecular data related to mitochondrial, oxidative and apoptotic pathways. All additional outcome measures not depicted here but reported in the included studies were also examined. In addition, next to the parameters of interest, information on adverse effects following the intervention were sought to provide a full picture of the current state of knowledge and subsequent risks. The units of measurement varied depending on the data extracted; e.g., if molecular data were extracted from enzymatic activities, units would be nmol of produced or reduced substrate per minute and mg of protein. 

The risk of bias was assessed by two review authors guided by the criteria recommended by the International Cochrane Collaboration: At least two external researchers were aware of the process, methodological approaches and follow-up of the study. To ensure the completeness of the outcome data, only treatment interventions using anti-apoptotic molecules in processes of neurodegeneration and neuroprogression were considered, mainly conducted in research animal models. Blinding intervention was conducted by two blinded researchers during the selection criteria and data extraction (Ryyan qcri). Different data storage (cloud and hardware) was used and access restricted to the researchers involved throughout the study. During the selective outcome reporting, primary and secondary outcomes were listed and reported, for further comparisons. Molecular and clinical findings were analyzed in order to elucidate the adequateness of the anti-apoptotic interventions in each specific context of neurodegeneration and/or neuroprogression. Any potential conflicts of interests were also explored. Any potential source of bias (risk of bias) was considered, such as bias towards favorable outcomes that may have occurred in the study. Finally, the assessment of publication bias was defined by identification of unpublished outcomes and studies, when available. Unpublished findings were searched via meeting abstracts tracked through Google Scholar, PhD theses available at the university repository, informal sources and, if needed, by contacting the authors of the studies included.

During the strategy for data synthesis, the approach plan was defined as follows: data were synthesized in a narrative manner and included studies were described according to: (i) the type of intervention, including in vivo or in vitro research of neurodegeneration and neuroprogression using anti-apoptotic molecules, and clinical trials; (ii) the type of pharmacological agent, including pan-caspase inhibitors, as well as specific anti-caspase-3 and anti-cytochrome c release; (iii) the characteristics of the study population and (iv) type of outcomes, including molecular parameters, such as neuroconnectivity, mitochondrial function and oxidative stress apoptotic markers and clinical findings of a global nature with special attention to secondary off-target reported events.

## 3. Results

**Figure 3:**
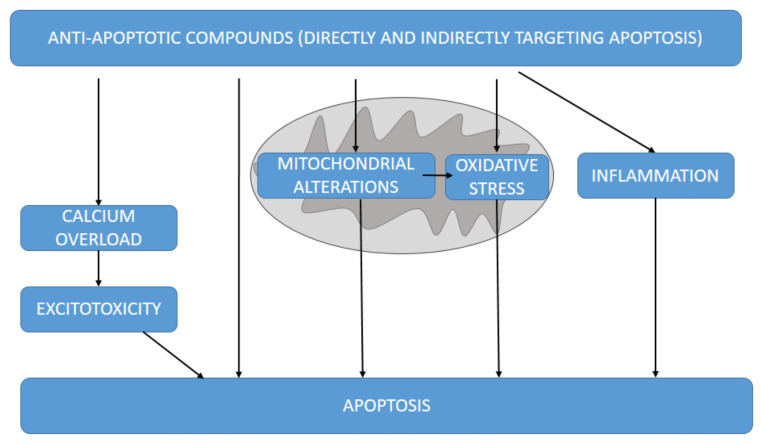
Different mechanisms by which the anti-apoptotic molecules herein discussed could target apoptosis, directly or indirectly through excitotoxicity, mitochondrial alterations and derived oxidative stress and inflammation.

A large number of anti-apoptotic molecules targeting apoptosis have been tested in different experimental neurodegenerative models. Most of them directly or indirectly target apoptosis through different mechanisms (Figure 3).

Some groups have suggested a neurodegenerative component of SZ [62]. In such cases, whether cell death is involved in the neuronal loss in neurodegenerative processes has important implications for the rational development of therapeutic strategies. The number of studies related to the use of specific anti-apoptotic molecules at the level of caspases in the classic neurodegenerative diseases (AD, PD, HD and ALS) vs. SZ is shown (Table S2). The outcomes indicate the lack of studies using apoptotic inhibitors in SZ with respect to those in classic neurodegenerative disorders. 

### 3.1. Apoptotic Alterations in Classic Neurodegenerative Disorders

Accumulating data suggest essential roles for apoptotic pathways in the pathophysiology of a spectrum of neuropathological disorders [63]. Increased neuronal apoptosis has been widely demonstrated in classic neurodegenerative disorders, often via grafted or β-amyloid-induced-neurodegeneration in AD models [44,64] through 1-methyl-4-phenyl-1,2,3,6-tetrahydropyridine-(MPTP)-induced-neurodegeneration in PD models [65], through 3-nitropropionic acid (NP3)- or malonate-induced neurodegeneration in HD models [46,66] and through superoxide dismutase-1 (SOD1)-mutations in ALS models [47,67]. 

Abnormal levels of apoptotic markers such as anti-apoptotic (Bcl-2/Bcl-xL) and pro-apoptotic proteins (Bax/Bak/Bad), Bcl-2 protein family members, initiator caspases-8 and -9 and the effector caspases-3 and -6 have been observed in experimental models of AD [68] and PD [69,70,71]. The key role of Bax in MPTP-induced-neurotoxicity is illustrated by the demonstration that mutant mice deficient in Bax are resistant to the induced toxicity of PD models [39]. Caspase-1 has also been implicated in other neurodegenerative contexts, such as HD [72]. Precisely in the context of HD, distinct studies investigated the harmful influence of human mutant huntingtin in the apoptotic cascade, specifically by triggering various Bcl-2 Homology 3 (BH3)-only proteins [73]. The functional role of caspase-1 and -3 has also been described in ALS [74]. Taking all these molecular events together, the etiopathogenesis of the four most representative classic neurodegenerative disorders is widely associated with the dysregulation of apoptotic pathways, similar to the case of SZ, for which ongoing apoptotic molecular alterations have been described in the previous section.

### 3.2. Intervention of Apoptotic Molecular Pathways in Neurodegenerative Disorders

The implication of caspases has been proposed as a common therapeutic target for multineurodegenerative disorders [75] and interfering at this molecular level has led to promising results. The broad-spectrum cysteine protease inhibitors (cathepsin, calpain and caspase) benzyloxycarbonyl-Val-Ala-Asp-fluoromethylketone (Z-VAD-FMK) [76], and peptide-inhibitors of caspases-2, -3 and -9 attenuated the loss of dopaminergic ventral midbrain cell bodies (but not neurites) [77]. Similarly, Z-VAD-FMK attenuated mutant SOD1-mediated cell death in transfected PC-12 cells and in transgenic SOD1 mice [74,78], and also resulted in delayed disease onset and mortality in these ALS animals [74]. Inhibiting caspase cleavage of huntingtin reduced toxicity and aggregate formation in neuronal and non-neuronal events [79]. Expression of a dominant-negative mutant form of caspase-1 in R6/2 mice extended survival and delayed the appearance of neuronal inclusions, receptor alterations and the onset of symptoms [72], and was associated with increased resistance to the neurotoxins used to model HD [80]. Pharmacological inhibition of particular members of the caspase family, such as caspases-2, -3, -8 and -12, protects against β-amyloid-induced apoptotic cell death in vitro [38,81]. Beyond caspases, but still sticking to anti-apoptotic interventions, overexpression of Bcl-2 protected dopaminergic cells against MPTP-induced neurodegeneration [82]. The tumor suppressor protein p53, which is activated after MPTP-intoxication [83], is one of the rare molecules known to regulate Bax expression [84]. p53-inhibitors attenuated MPTP- induced Bax upregulation and the degeneration of dopaminergic neurons [85], and p53-null mice were resistant to MPTP-induced death of dopaminergic neurons (Table S3). 

### 3.3. Mitochondrial Anti-Apoptotic Targets against Neurodegeneration

The fact that apoptosis is mostly mediated by mitochondria explains why most previously mentioned intervened apoptotic molecules are mitochondrially-related. Bcl-2 is located in the mitochondrial outer membrane, whereas cytoplasmic bax is translocated to the mitochondria upon induction of cell death, and cytochrome-c and caspase-9 are released by mitochondria [86]. Accordingly, most anti-apoptotic targets halting neuronal damage found in the literature are aimed at mitochondria or are mitochondrially-driven (Tables S4 and S5). Inhibition of cytochrome-c release was associated with therapeutic benefits in HD mice [40]. Overexpression of Bcl-2 mitigated neurodegeneration in both in vitro and in vivo models of ALS [78] and have been shown to prolong survival in the classic model of the disease consisting of transgenic SOD1 mice [41]. The carbonic anhydrase-inhibitor methazolamide, previously related to therapeutic events in HD models, prevented β-amyloid-induced mitochondrial dysfunction and caspase activation, protecting neuronal and glial cells in vitro and in vivo [87]. Microarray analysis revealed that the cyclin-dependent kinase olomoucine promoted downregulation of the protein E1B 19-kDa-interacting protein 3 (BNIP3), a pro-apoptotic Bcl-2 family protein involved in mitochondrial disruption in lipopolysaccharide and nitric oxide (NO)-cell death induced microglial cells [88]. On the other hand, mitochondrial dysfunction promotes oxidative stress which, in turn, may precede apoptosis, thus oxidative damage is an interesting target in neurodegeneration. To date, mitochondrially targeted molecules against oxidative stress is still one of the most effective therapeutic strategies in neurodegenerative disorders [40,89,90], with the presentation of a wide array of molecules (Tables S4 and S5). Anti-apoptotic-related natural compounds and antioxidants have also shown therapeutic effectiveness in HD [91]. Withanolides exerted beneficial effects on cognitive functions and ultimately neuroprotective effects in HD models [90]. Interestingly, withanolide A promoted neuritic regeneration and synaptic reconstruction in other neurodegeneration models [92]. BN-82451, a newer antioxidant, improved motor ability and attenuated neurodegeneration in a mouse model of HD [90,93]. Vitamin C and α-lipoic acid also had beneficial effects on motor symptoms and extended survival rates in rodents [93]. Coenzyme Q10, a component of mitochondrial membranes and a free radical scavenger, which has also been associated with anti-apoptotic effects [94], presents therapeutic effects in HD models. Antioxidants have also been tested in the psychiatric field. Astaxanthin has been related to antidepressant-like effects in different experimental models, including AD and PD [95]. Importantly, nicotinamide, a precursor of the coenzymes NAD and NADP involved in energy metabolism via redox reactions, protects against ketamine-induced apoptotic neurodegeneration in the infant rat brain [96], which could mimic a prodromal stage of SZ [96]. 

Herein we provide quantitative data of mitochondrial outcomes related to the administration of anti-apoptotic compounds, indicating less oxidative stress biomarkers in AD [97], PD [98] and HD [99], increased mitochondrial biogenesis and ATP levels in PD [100] and increased mitochondrial respiration in AD [101], among others (Table S6).

### 3.4. Other Anti-Apoptotic-Related Molecular Pathways Associated with Neuroprotection

Often, also related to oxidative stress, hormone metabolism plays an important role in neurodegenerative processes. This is in line with the classical use of hormone withdrawal as a model of neurodegeneration [102]. Melatonin, the circadian hormone with antioxidant features, reversed H-89 induced spatial memory deficit with involvement of oxidative stress and mitochondrial function [103]. Recent clinical trials with melatonin in PD have not led to conclusive results as yet [104], although previous studies showed a significant improvement in clinical global impression (CGI: 6.1 versus 4.6; p= 0.024) [105]. Neuroprotection by estrogen takes place in the brain, and the mitochondrial compartment is considered the presumed therapeutic target [106]. 17β-Estradiol protected retinal nerve cells against H_2_O_2_-induced apoptosis by significantly inhibiting Bax-involved mitochondrial apoptosis via the activation of the protein kinase (AKT) signal pathway [107]. Hormones involved in glucose metabolism, known to be disrupted in comorbidity with several neurodegenerative processes, have also been associated with anti-apoptotic properties and improvement of several neurodegenerative features [108], and there is even a clinical trial in PD patients that showed promising initial results [109]. The neuroprotective effects of hormonal interventions go far beyond the molecular level, leading to the improvement of neurodegenerative clinical manifestations associated with the intervention of apoptosis [110]. 

In summary, the wide array of aforementioned caspase-inhibitors, mitochondrial, antioxidant and hormonal molecules included in this study interact with distinct biological pathways and have been associated with anti-apoptotic properties in neurodegeneration. The multitarget effects of such compounds underlie the pharmacological mechanisms of action to tackle neurodegeneration by converging in cell death. This common anti-apoptotic link is the requirement for their inclusion in this study, and the specific mechanisms of action against cell death have been tested in several models of neurodegeneration (Table S3). This is in line with the wide array of compounds that have been studied to treat the quadriad neurodegenerative disorders by interfering with apoptosis [111] at both the mitochondrial level (Table S4) and through the interaction with a variety of targets at distinct cell death signaling pathways (Table S5). 

Both representative, qualitative (Table S5) and quantitative (Table S6) outcomes using apoptotic-inhibitors in neurodegenerative models, including AD, PD, HD and ALS have been summarized. First, quantitative data of molecular outcomes showed significant improvement of synaptic function in mice [112] and decreased neuronal death in neuroblastoma in AD [113] increased neuronal survival in PD neuroblastoma [114], HD [46] and ALS rodents [115], increase of neurotransmitters [116] and decreased proinflammatory cytokines in PD rodents [117], among others. Importantly, significant quantitative findings were not restricted to molecular data but also to symptom improvement, since quantitative clinical outcomes showed significant amelioration of behavioral parameters assessed in different neurodegenerative models including AD mice [72], HD rats [109,118], as well as time of survival, time before disease onset or disease progression in ALS mice [115], upon applying anti-apoptotic approaches (Table S6). 

### 3.5. Targeting Apoptosis in SZ

Despite the involvement of apoptotic pathways in SZ and the evidence of anti-apoptotic therapeutic effectiveness in the classic neurodegenerative disorders, the number of studies investigating anti-apoptotic molecules is lacking in SZ (Table S2). Currently, the positive symptoms of SZ are clinically attenuated through a known battery of antipsychotics. Interestingly, certain second-generation antipsychotics could have a neuroprotective role in vitro via an anti-apoptotic action, as demonstrated in a previous study from our group using a neuroblastoma cell model. The results indicated that haloperidol induces apoptosis, while risperidone and paliperidone may afford protection against it [119]. Both olanzapine and clozapine present antioxidative and anti-apoptotic activities [120]. Accordingly, antipsychotics have been related to functional changes in mitochondria as the main apoptotic orchestrator. Clozapine has been specifically reported to improve mitochondrial function by altering mitochondrial membrane potential [121]. Conversely, haloperidol, a first-generation antipsychotic, is linked to destructive changes in mitochondria [90,121]. A recent study also found that clozapine may have a neuroprotective effect on adult neural stem cells from ketamine-induced cell death in correlation with decreased apoptosis [122]. 

Other widespread pharmacological options, beyond antipsychotics and related to anti-apoptotic mechanisms, have been associated with neuroprotection in the context of SZ at the mitochondrial level (Table S4), mainly as adjunctive therapies in patients. Adjuntive N-acetylcysteine has been reported to reduce negative and general symptoms in SZ patients in several clinical trials [123]. N-acetylcysteine was associated with neuroprotective effects and prevented apoptosis mediated signals in various rodent models underlying mitochondrial malfunctioning in SZ pathology [121]. It protects against cadmium-induced ROS toxicity marked by reduced mitochondrial membrane potential, reduced Bcl-2 expression and p53 expression and a reduction in the caspase pathways [121].

Despite the lack of studies using caspase and apoptotic-inhibitors in SZ (Table S2), a limited proportion of previously expounded substances associated with anti-apoptotic mechanisms have been tested in this psychiatric disease (Table S7). Melatonin has been reported both as a surrogate marker and therapeutic agent in SZ [124]. SZ-like behavior was reported to be unchanged by melatonin supplementation in rodents [125], whereas other studies report attenuation of SZ-like symptoms and a protective effect on the prefrontal cortex region of brain by mitigating the alteration of neurotoxicity markers [126]. Another double-hit compound in SZ, considered both a surrogate marker and a therapeutic approach, is retinoic acid [127]. Retinoic acid, described to protect against proteasome inhibition-associated cell death in neuroblastoma cells via the survival protein kinase B (AKT) pathway [128], has been used as an add-on with antipsychotic treatment and showed a significant reduction of positive symptoms in SZ patients [129]. Finally, other adjunctive therapies with antipsychotics have also been tested with other anti-apoptotic-like substances, including estradiol, quercetin and erythropoietin, among others, all leading to promising outcomes [130,131,132] (Table S7). However, the experience using anti-apoptotic molecules exclusively rather than as an adjunctive treatment in SZ patients is null.

## 4. Discussion

Despite the high number of studies and growing evidence of the effectiveness of anti-apoptotic therapeutic strategies in classic neurodegenerative disorders, there is very little research on anti-apoptotic targets in SZ. This would likely be explained by the limited investment in the psychiatric field, even in developed countries [133], rather than by the lack of scientific evidence of the potential positive effects of these surrogate anti-apoptotic therapies in SZ. Considering both the neuroprogressive and dendritic apoptotic nature of SZ [20,62], through excessive synaptic pruning derived from glutamatergic storm and associated excitotoxicity, the use of apoptotic-inhibitors in SZ is likely to be a promising approach. This study arose as a proposal for the use of anti-apoptotic drugs and their potential therapeutic effect in SZ. In summary, experimental SZ models via ketamine and MK801 induction, interacting with GABA/NMDA receptors, promote subsequent apoptotic molecular pathways underlying the symptomatology of SZ. Such molecular events, related to neuronal death, may be inhibited by anti-apoptotic compounds that eventually protect against excessive synaptic pruning in the SZ brain, preventing the clinical symptoms of the disease. Specifically, anti-apoptotic molecules inhibit ROS production and promote mitochondrial membrane potential maintenance, mainly by blocking several key elements of the apoptotic cascade, including cytochrome-c release or caspase activation, ultimately resulting in protection against the accelerated dendritic apoptosis seen in SZ. The scientific evidence for the therapeutic effectiveness of anti-apoptotic strategies in neurological disorders is striking and goes far beyond the data presented herein. Targeting programmed cell death in ischemia/stroke [134] has been a common strategy which has not been considered in this study, as such mechanical models are most likely far from the molecular neurodevelopmental triggers in SZ and, subsequently, fall outside the scope of this review. 

Targeting cell death is not only useful as a novel therapeutic option but also to elucidate the etiopathogenic basis of the disease. Therefore, by the use of X-linked apoptotic inhibitor (XIAP), a new relevant role of caspase-9 was elucidated in ALS [135]; or, by using the pan caspase inhibitor Z-VAD-FMK, a new role for caspase activation as a potential route rather than an obligatory initiation step of tubulin associated unit (TAU) aggregation was defined in AD [136]. The fact that the molecular basis of SZ is not fully established gives rise to another argument encouraging the use of anti-apoptotic molecules to further shed light on the unknown molecular nature of this disease.

The available anti-apoptotic approaches with great potential to restore biological pathways do not only include anti-caspase molecules, but also other apoptotic targets that could actively intervene, such as calpains [137] or cathepsins [138], among others. Upstream targets at the mitochondrial level deserve special attention [139], as well as antioxidative strategies, since they target oxidative damage preceding subsequent apoptosis [140]. Such is the case of N-acetyl-cysteine that has been tested in SZ leading to promising results [141,142]. The phosphodiesterase-type-5-inhibitor sildenafil modulated the expression of pro- and anti-apoptotic proteins of the extrinsic and intrinsic pathways and promoted remyelination in the spinal cord [143], indicating neuroprotective effects in a placebo-controlled study on cognition in SZ [144]. Thus, this review has considered multitarget molecules ultimately targeting cell death while hypothesizing that an anti-apoptotic prodromal and upstream intervention via bax inhibition and cytochrome-c release would prevent SZ-like symptoms.

There is evidence of the interaction of antipsychotic drugs with the apoptotic pathways [120]. The dopamine-stabilizer pridopidine protected cells from apoptosis, and resulted in highly improved motor performance in the HD model using R6/2 mice [145]. Chlorpromazine, an antipsychotic agent, induces G2/M phase arrest and apoptosis via regulation of the PI3K/AKT/mTOR-mediated autophagy pathways in human oral cancer [146]. Prototypical antipsychotic drugs protect hippocampal neuronal cultures against cell death induced by growth medium deprivation [147]. Additionally, the literature also reports a close interaction between some drugs altering neurotransmitter levels and mitochondria [148]. In contrast to these neuroprotective effects of some antipsychotics, haloperidol has been recently found to present pro-apoptotic features [149], which, in a paradoxical manner, would eventually aggravate the disease. Interestingly, erythropoietin prevented haloperidol-induced neuronal apoptosis through the regulation of brain-derived neurotrophic factor (BDNF) [150], and protective effects against haloperidol-toxicity have also been reported for cystamine [151]. Regardless of such controversial anti- or pro-apoptotic effects associated with different antipsychotic drugs depicted in the literature, the antipsychotic-apoptotic relationship suggests that the apoptotic pathways are indeed involved in the course of SZ and, therefore, in line with the rationale of the present study, anti-apoptotic strategies could serve as the optimal therapeutic option for SZ. However, adequate therapeutic drugs targeting apoptosis that allow us to more effectively halt, prevent or revert the development of SZ are yet to be discovered. In addition, as reviewed, the current anti-apoptotic approaches in SZ have been mainly tested as adjunctive treatments included within antipsychotic schedules rather than as exclusive interventions in SZ patients [129], probably due to the lack of preliminary in vitro and in vivo research studies.

From the main co-treatment of anti-apoptotic approaches combined with antipsychotics reported in SZ, minocycline is probably one of the most representative candidates. This antibiotic with anti-apoptotic effects has been widely studied in different neurological disorders including AD [152], PD [153], HD [154] and ALS models [155], among others, leading to promising findings in most cases [156]. The anti-apoptotic action of minocycline consists in the inhibition of cytochrome-c release from mitochondria by attenuating the mitochondrial permeability transition pore and inhibiting caspase-1 and -3 [153]. Minocycline inhibited Aβ-fibril formation [152], attenuated amyloid-induced microglial activation [157] and reduced the inflammatory events associated with the prevention of cognitive deficits. Minocycline has also been used in several clinical trials in the main neurodegenerative disorders, including AD, although, in the most recent clinical trial, it did not delay the progress of cognitive or functional impairment in people with mild AD during a two-year period [158]. Minocycline administration has also been associated with beneficial effects in HD [93] and related to delayed mortality in a transgenic mouse model of HD [159] and of ALS [155]. Accordingly, it is one of the few anti-apoptotic approaches conducted in SZ [160]. While some studies do not report any improvement associated with minocycline administration and failure of inhibition of cytochrome-c release [101], others report promising findings preventing neurodegeneration [153,156]. Controversial data have been found for the use of minocycline in SZ as well. Whereas some studies do not support a therapeutic role of minocycline in SZ [161], others cautiously report that minocycline may be helpful in treating negative and cognitive symptoms in SZ [162]. Minocycline administration has been associated with an amelioration of cognitive deficits and correlated with the remission of negative symptoms and reduction of inflammatory parameters [163]. Robust clinical improvements with minocycline treatment have also been described via the Positive and Negative-Syndrome-Scale (PANSS) for SZ [164]. Controversial data regarding minocycline administration extends to other molecules with anti-apoptotic properties in SZ. SZ-like behavior was not altered by melatonin supplementation in rodents [125]. However this compound has been reported to improve sleep disturbance, antipsychotic side effects and benzodiazepine discontinuation in SZ [124]. Together, these data demonstrate the scarcity of conclusive data and the lack of research targeting apoptotic molecules in SZ, underpinning the urgent need to further investigate this unexplored field.

When proposing anti-apoptotic therapeutic targets in SZ, special attention should be addressed to potential side effects related to such treatments, especially those concerning teratogenesis, carcinogenesis and cytotoxicity, considering their ability to intercede in cell death. Most of the studies conducted in in vitro and in vivo experimental interventions, herein reviewed, mainly investigate molecular events related to the drug-derived mechanism of action, without reporting any off-target effect [165,166]. On one hand, the FMK group of general cysteine protease inhibitors (cathepsin, calpain and caspase) cause irreversible enzyme inhibition making them unsuitable for clinical use. However, interestingly, most of the anti-apoptotic interventions addressed in other neuropathological contexts are commercially available and, therefore, their adverse secondary events have already been tested. Minocycline clinical trials demonstrated adequate tolerability and did not report adverse events after two years of administration [154]. Memantine, used in AD [167,168], has been mainly related to headache, constipation, sleepiness, and dizziness and severe side effects may include blood clots, psychosis, and heart failure [167]. High doses of methylene blue (>10µM) have been associated with cytotoxic effects in vitro [169]. It seems that all proposed uses of methylene blue entail levels that block monoamine oxidase, so cessation of serotonin reuptake inhibitors should be very carefully considered before using methylene blue [170]. Cytoprotective effects and cytochrome-P450 toxicity has been studied in a library of anti-apoptotic compounds leading to some safe molecules such as selective, reversible, small-molecule caspase-3-inhibitor (RBC1023) [171].

It is important to emphasize the challenging nature of anti-apoptotic interventions in neurological contexts. Numerous pharmacological compounds have been investigated due to their potential ability to reduce neuronal injury. Despite promising data from laboratory work, most of these agents presented disappointing clinical results. This is mainly due to the complex mechanisms involved in neuronal injury and the difficulty in controlling physiological factors, probably hampering the availability of a “perfect model of the disease” (current SZ models may be far from the real biological processes underlying the disease). One of these complexities relies on the time framework in which both the investigations and the interventions are conducted. It is most likely that prodromal interventions represent the most effective therapeutic option for SZ, before the apoptotic cascade leading to irreversible excessive synaptic pruning occurs. Moreover, heterogeneity of SZ could impact on the therapeutic efficacy of anti-apoptotic drugs when considering the loss of dendritic spines in the prefrontal cortex, while other brain regions present opposite results (such as dorsal and ventral striatum, where there are more synapses and spines). The combination of multiple strategies, including prodromal intervention, especially in patients with prominent deficit symptoms, and the use of compounds targeting different apoptosis-related pathways and the control of physiological variables, may afford the most meaningful results focused on neuroprotection in SZ.

## Data Availability

Not applicable.

## Supplementary Materials

The following supporting information can be downloaded at: https://www.mdpi.com/article/10.3390/antiox11112275/s1, Figure S1: Flow chart of the studies included in this review; Table S1: In vitro and in vivo models using Bax-/Bax- genetic manipulation, underpinning the relevance of mitochondrial apoptotic targets in the therapeutic approaches for neurodegeneration; Table S2: Number of studies using apoptotic inhibitors in the classic neurodegenerative disorders including AD, HD and PD vs. SZ; Table S3: In vivo and in vitro approaches against apoptosis in several neurodegenerative experimental models.; Table S4: Mitochondrially-targeted drugs in neurodegenerative and other neurological disorders; Table S5: Neuroprotective substances targeting cell death at distinct molecular levels in different neuropathological models; Table S6: Quantitative data of molecular and clinical outcomes by using anti-apoptotic compounds in models of neurodegeneration and neuroprogression; Table S7: Therapeutic approaches against SZ with compounds indirectly targeting apoptosis. References [39,40,72,78,85,90,96,97,98,101,103,108,111,112,113,115,117,118,128,130,132,135,137,139,140,143,144,145,151,155,163,166,171,172,173,174,175,176,177,178,179,180,181,182,183,184,185,186,187,188,189,190,191,192,193,194,195,196,197,198,199,200,201,202,203,204,205,206,207,208,209,210,211,212,213,214,215,216,217,218,219,220,221,222,223,224,225,226,227,228,229,230,231,232,233,234,235,236,237,238,239,240,241,242,243,244,245,246,247,248,249] are cited in the supplementary materials.
