# Supplementary material for: Systematic Review of the Therapeutic Role of Apoptotic Inhibitors in Neurodegeneration and Their Potential Use in Schizophrenia
Source: Antioxidants (Basel). 2022 Nov 17;11(11):2275. doi: 10.3390/antiox11112275 (PMC9686909; doi:10.3390/antiox11112275)
Supplement: Supplementary file 1 [file antioxidants-11-02275-s001.zip › antioxidants-1947631-supplementary.pdf]

antioxidants

Supplementary Material

Supplementary Figure S1. Flow chart of the studies included in this review.

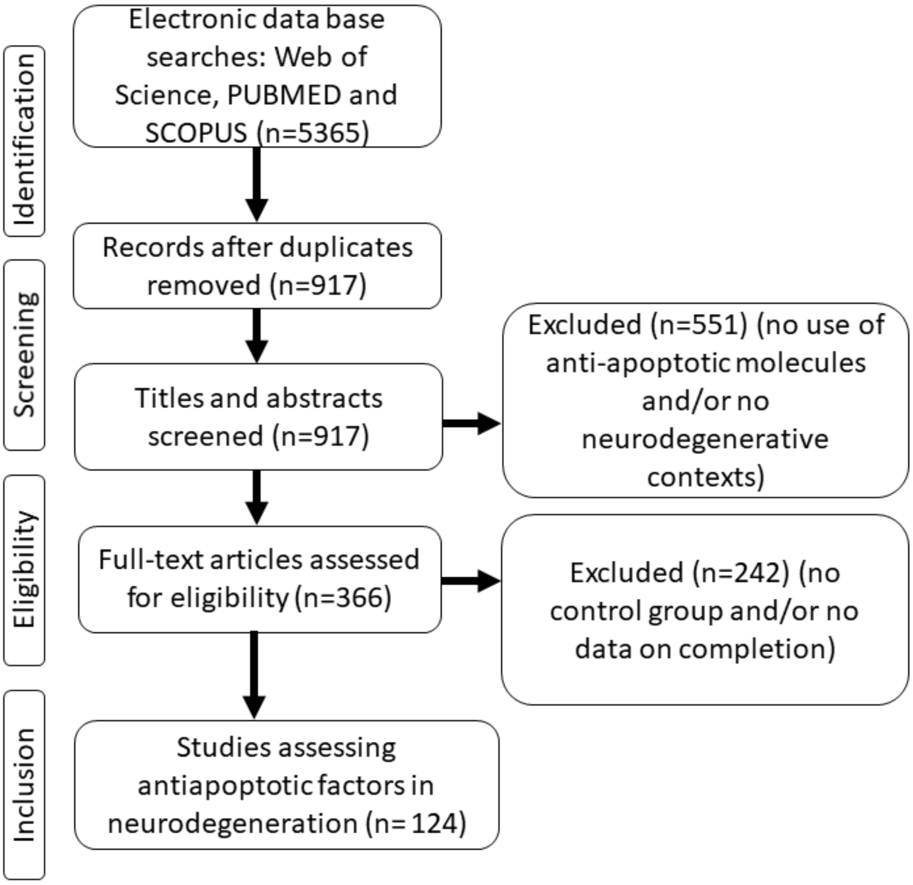

Supplementary Table S1. In vitro and in vivo models using Bax-/Bax- genetic manipulation, underpinning the relevance of mitochondrial apoptotic targets in the therapeutic approaches for neurodegeneration.

**Bax-/bax- *in vitro* and *in vivo* models**			
**Pathology**	**Model**	**Conclusions**	**Reference**
Neurodegeneration	Knockout mice	A significant reduction of astrocyte density is observed in the hippocampus of Bax knockout mice	[1]
Neurodegeneration (excitotoxicity)	Oxygen/glucose deprivation in Bax-deficient neurons	The ability of Bax to support dynamic ER Ca2+ handling is critical for cell death signaling during periods of neuronal overexcitation	[2]
Unfolded protein response	ER stress induced in neurons	ER stress commits neurons to die before cytochrome c release and this commitment requires Bax activation and c-jun N-terminal kinase signaling	[3]
Neurodegeneration	Apoptosis induced in sympathetic neurons	Puma provides a critical link between p53 and Bax, and is both necessary and sufficient to mediate DNA damage-induced apoptosis of sympathetic neurons	[4]
Parkinson's disease	MPTP mice	Mutant mice lacking Bax are significantly more resistant to MPTP than their wild-type littermates	[5]
Neurodegeneration	Radiation induced in hippocampal neurons	P53 and Bax are necessary for radiation-induced cell death in postnatal cultured hippocampal neuron (caspase-independent manner)	[6]
Neurodegeneration	Apoptosis induced in cerebellar granule cells	Bax deleted granule cells do not undergo apoptosis (during potassium induction), yet do undergo an excitotoxic cell death in response to stimulation with NMDA	[7]
Neurodegeneration	Trophic factor deprivation	Bax-deficient superior cervical ganglia and facial nuclei possess increased numbers of neurons	[8]

ER, endoplasmic reticulum; MPTP, 1-methyl-4-phenyl-1,2,3,6-tetrahydropyridine; NMDA, N-Methyl-D-Aspartate; SOD, superoxide dismutase.

Supplementary Table S2. Number of studies using apoptotic inhibitors in the classic neurodegenerative disorders including AD, HD and PD vs. SZ.

MOLECULE	CAS No.	Target/action	TNS	TNS AD	TNS HD	TNS PD	TNS SZ
Ac-DEVD-CHO	169332-60-9	Caspase-3	53	1	2	9	0
Ac-FLTD-CMK	2376255-48-8	Caspase-5, -11	1	0	0	0	0
Aristolactam I	13395-02-3	Caspase-3	56	1	0	0	0
Asperosaponin VI	39524-08-8	Caspase-3	68	1	0	0	0
Belnacasan (VX-765)	273404-37-8	Caspase-1, -4	8	3	0	1	0
Biotin-VAD-FMK	1135688-15-1	Pan caspase	1	1	0	0	0
BOC-D-FMK	634911-80-1	Pan caspase	81	1	1	1	0
Chelidonic acid	99-32-1	NF-κB, Caspase-1	26	1	0	0	0
Crustecdysone (20-Hydroxyecdysone)	5289-74-7	Pan caspase	1839	1	3	7	0
Duocarmycin A	118292-34-5	Caspase-3, -9	85	1	0	0	0
Emricasan (PF 03491390)	254750-02-2	Pan caspase	54	1	0	1	0
EP1013	223568-55-6	Pan caspase	28	1	0	1	0
Ivachtin	745046-84-8	Caspase-3	477	1	0	0	0
ML132 (NCGC 00185682)	1230628-71-3	Caspase-1	4	1	0	1	0
Ossirene	106566-58-9	Caspase-1	1	1	0	0	0
Paris saponin VII	68124-04-9	Caspase-3, -9 and Bcl-2	19	1	0	0	0
Penicillic acid	90-65-3	Fas ligand, Caspase-8	271	1	0	0	0
Pralnacasan	192755-52-5	Caspase-1	19	1	0	0	0
Q-VD-OPh	1135695-98-5	Pan caspase	144	4	1	1	0
Senkyunolide I	94596-28-8	Caspase-3	93	1	0	0	0
Sesamolin	526-07-8	JNK, p38, MAPKs and caspase-3	108	1	0	1	0
Tauroursodeoxycholate	14605-22-2	Caspase-3, -12	1036	1	0	20	0
Tauroursodeoxycholate Sodium	35807-85-3	Caspase-3, -12	*90	1	0	1	0
Thevetiaflavone	29376-68-9	Bcl-2, Bax and Caspase-3	4	1	0	0	0
Wedelolactone	524-12-9	Caspase-11	164	1	0	1	0
Z-VAD(OMe)-FMK	187389-52-2	Pan caspase	61	1	1	0	0
Z-VAD-FMK	161401-82-7	Pan caspase	3499	31	10	27	0
Z-DEVD-FMK	210344-95-9	Caspase-3	755	21	4	13	0
Z-LEHD-FMK	210345-04-3	Caspase-9	283	1	0	2	0
Z-IETD-FMK	210344-98-2	Caspase-8	353	1	0	1	0
Z-YVAD-FMK	210344-97-1	Pan caspase	68	1	0	0	0
Z-WEHD-FMK	210345-00-9	Caspase-3, -12	7	1	0	2	0
ZYZ-488	1470302-79-4	Caspase-3, -9	2	1	0	0	0
Z-Asp-CH2-DCB	153088-73-4	Pan caspase	90	1	0	0	0
Z-LEHD-FMK TFA	524746-03-0	Caspase-9	3	1	0	0	0

TNS, Total number of studies; AD, Alzheimer's disease; HD, Huntington's disease; PD, Parkinson's disease; SZ, Schizophrenia. NOTE: Etiopathogenic studies have been included. *A clinical trial with anti-apoptotic sodium phenylbutyrate-taurursodiol has been recently conducted on amyotrophic lateral sclerosis leading to promising results in the slower functional decline of the patients (155.)

Supplementary Table S3. In vivo and in vitro approaches against apoptosis in several neurodegenerative experimental models.

Disease	Model	Manipulation	Pharmacological inhibition	References
Alzheimer's disease	Aβ Hippocampal neurons	Aβ aggregates	Rhizolutin (caspase-3)	[9]
	Transgenic mice	TgCRND8*	QVD-OPh (pan caspase)	[10]
Parkinson's disease	Transgenic mice	Bax knockout	Pifithrin-alpha/Z-1-117 (p53)	[11], [12]
		Bcl-2 transgenic	CEP-11004 (JNK)	[13]
		P53 knockout	Minocycline (cyt C, caspase-1, -3)	[14]
	Induced mice	MPTP- and 6-OHDA-induced	Z-DIPD-FMK (caspase-3)	[15]
	Dopaminergic neurons	Alpha-synuclein	QVD-OPh, zVAD-FMK/M-920, zVDVAD-FMK (caspase-2), zDEVD-FMK/M-725 (caspase-3), zLEHD-FMK (caspase-9)	[16]
Huntington's disease	R6/2 transgenic mice	Caspase-1 transgenic	zVAD-FMK, minocycline (cyt c, caspase-1, -3)	[17]
	BACHD transgenic mice	BACHD transgenic	ED11 (caspase-6)	[18]
Amyotrophic lateral sclerosis	SOD1 transgenic mice	Bcl-2 transgenic	zVAD-FMK	[19]
		Caspase-1 transgenic	Minocycline (cyt C, caspase-1, -3)	[20]
		G93A-SOD1	JGK-263 (Glycogen synthase kinase-3β)*	[21]

6-OHDA, 6-hydroxydopamine; BACHD, bacterial artificial chromosome (BAC)-mediated transgenic mouse model; Cyt c, Cytochrome c; ED11 peptide (GRKKRRQRRRPPQSSEIVLDGTDN); FasL, Fas Ligand; Glycogen synthase kinase-3β is a critical activator for neuronal apoptosis; JNK, JUN-N-terminal kinase; MPTP, 1-methyl-4-phenyl-1,2,3,6-tetrahydropyridine; QVD-OPh, quinolyl-valyl-O-methylaspartyl-[-2, 6-difluorophenoxy]-methyl ketone; SOD1, Superoxide dismutase; zDEVD-FMK, benzyloxycarbonyl-Asp(OMe)-Glu(OMe)-ValAsp(OMe)-fluoromethylketone; zLEHD-FMK, benzyloxycarbonyl-Leu-Glu(OMe)-His-Asp(OMe)-fluoromethylketone; zVAD-FMK, benzylozycarbonil-Val-Ala-Asp-fluoromethylketone; zVDVAD-FMK, benzyloxycarbonyl-Val-Asp-Val-Ala-Asp-fluoromethylketone

Supplementary Table S4. Mitochondrially-targeted drugs in neurodegenerative and other neurological disorders.

Drug class (Compound)	Mechanism of action	Therapeutic outcomes	References
a-Lipoic acid	Scavenges the toxic by-products of lipid peroxidation	Antioxidant properties in AD	[22], [23]
Carotenoid (Lycopene)	Suppress oxidative damage	Antioxidant, anti-inflammatory, memory enhancing and neuroprotective activities in HD	[23], [24]
Cholest-4-en-3-one	Scavenges the toxic by-products of lipid peroxidation	Effective in treating painful diabetic and chemotherapy-induced neuropathies	[23]
Clozapine	Improves function of mitochondria by altering mitochondrial membrane potential	Neuroprotective properties in SZ	[23], [25]
Curcumin (volatile oil) (Curcuma longa)	Suppress tumor necrotic factor (TNF) activity, formation of Aβ plaques and protects brain cells from noxious agents	Antioxidant, anti-inflammatory and amyloid disaggregating properties in AD	[23], [24], [26]–[28]
Dichloroacetate	Activates the pyruvate dehydrogenase complex and lower cerebral lactate amounts	Neuroprotective activity in HD	[23], [29]
Disaccharide (Trehalose)	Inhibits amyloid formation, aggregation of β-amyloid and autophagic activities against aggregation proteins (huntingtin)	Neuroprotective properties in HD	[23], [24]
Epigallocatechin-3-gallate (Camellia sinensis)	Stabilize mitochondrial functions like ATP levels	Antioxidant properties in AD	[23], [24], [26]
Ferulic acid (Smallanthus sonchifolius)	Neuroprotective effect against oxidative stress and cell death induced by Aβ42 oligomers	Antioxidant properties in AD	[23], [24], [26]
Ginsenosides Rg1 and Rg3 (Ginseng)	Suppress Aβ induced neurotoxicity, Aβ associated generation of ROS and cell death	Neuroprotective effect in AD	[23], [24], [26]
Melatonin	Direct scavenger of many ROS species such as free radicals, peroxylnitrites, hydroxyls, peroxyls, and other nitrous oxides under normal conditions	Antioxidant properties in PD	[30]
Mitoquinone	Produces direct antioxidant action by scavenging peroxyl, peroxynitrite and superoxide ROS	Antioxidant properties in PD	[31]
N-acetylcysteine	Protects against cadmium induced ROS toxicity marked by reduced mitochondrial membrane potential, high cytoplasmic cytochrome c release, reduced Bcl-2 expression, p53 expression and caspase pathways	Neuroprotective properties in SZ	[23], [25]
Naringin, hesperidin and kaempferol (flavonoids)	Exert protective action against peroxynitrite induced oxidative damage and inhibit nitric oxide synthase (involved in HD)	Anti-inflammatory, antioxidant and neuroprotection in HD	[23]
Olanzapine	An antipsychotic agent with affinity for D1 and D2 dopamine receptors as well as 5-HT2A serotonin receptors	Improved motor symptoms in HD	[23]
Olesoxime	Scavenges the toxic by-products of lipid peroxidation	Antioxidant and neuroprotective activities in neuropathy	[32]
Perampanel (Celastrus regelii)	Inhibit nitric oxide synthase (involved in PD and HD)	Anti-inflammatory, antioxidant and neuroprotective activities in PD and HD	[23], [24]
Riluzole	Reduces ROS generation via induction of glutathione production	Antioxidant properties in ALS	[33]
Sesamol (Sesamum indicum)	Suppress inducible nitric oxide synthase (iNOS) expression and neuroinflammation in hippocampus neurons	Antioxidant, and neuroprotective activities in HD	[23]

Steroidal lactones (withaferin A, withanolide A, withanolide D-P) (Withania somnifera)	Improves cognitive functions and restores acetyl cholinesterase enzyme activity	Antioxidant and neuroprotective properties in HD	[23]
Terpene lactones (ginkgolides and bilobalides) and flavonoids (flavonols and flavone glycosides) (Ginkgo biloba)	Stabilize mitochondrial functions like ATP levels and interacts with mitochondrial electron transport chain	Antioxidant and neuroprotective properties in dementia, AD and PD	[23], [24], [26]
Triterpene saponin (glycyrrhizin) and phenol (isoliquiritigenin) (Glycyrrhiza)	Reduces oxidative stress and damage to brain cells	Antioxidant, anti-inflammatory and neuroprotective properties in dementia, AD and PD	[23], [24], [26]
Triterpenoid saponins (Bacosides A and B) (Herpestis monniera)	Scavenging of free radicals and improves memory	Antioxidant, anti-stress, antidepressant and useful in HD treatment	[23], [24]
Triterpenoid saponins (asiaticoside, asiatic acid and madecassoside) (Centella asiatica)	Reduction in the activity of electron transport chain enzymes and decreased mitochondrial viability	Antioxidant and neuroprotective properties in HD	[23]
Vitamin C	Maintains the integrity of cellular membranes in mitochondria	Antioxidant and neuroprotective activities in neuropathy	[23]
Vitamin E	Maintains the integrity of cellular membranes in mitochondria	Antioxidant properties in AD	[22], [23]
Z-LEHD-FMK	Caspase-9 inhibitor	Neuroprotective properties in epilepsy	[23]
Z-FA-MK	Anti-apoptotic in function which suppress some form of apoptosis	Effective therapeutic target in MS	[23], [34]

Supplementary Table S5. Neuroprotective substances targeting cell death at distinct molecular levels in different neuropathological models.

Substance	Target/Interaction	Disorder	*In vitro*	*In vivo* (animal/tissue)	Conclusions	Reference
Ac-YVAD-CMK/Ac-DEVD-CMK	Caspases and CPP32-like caspases	PD	-	rats (brain)	Failure of these caspase inhibitors to augment TH-ir neuron survival	[35]
		ND	Neuronal cell cultures	rats (blood, brain)	Rescued neuronal cells from cell death in response to oxidative stress and oxygen/glucose deprivation	[36]
Bacopa monnieri extract*	Oxidative stress	PNS	Amygdala	rat (brain)	Ameliorates Learning and Memory Impairments through Synaptic Protein, Neurogranin, Pro-and Mature BDNF Signaling, and HPA Axis	[37]
Cryptotanshinone (quinoid)	Oxidative stress and inflammation	PD	hiNPS	-	Anti-apoptotic properties in PD-hiNPCs, significantly reduced cellular apoptosis through mitochondrial restoration (reactive oxygen species and mitochondrial membrane potential). These effects are mediated via the nuclear factor erythroid 2-related factor 2 (NRF2) pathway in PD-hiNPCs.	[38]
Cystamine (CYS)	Oxidative stress/apoptosis	SZ	-	mice (brain)	Increased BDNF protein levels in mouse frontal cortex, prevention of chronic HAL treatment-induced reduction in BDNF, GSH, and Bcl-xl protein levels, prevention of reduction in neuronal cell viability, BDNF protein levels and apoptosis.	[39]
DHA (fatty acid)	Oxidative stress, modulation of membrane fluidity	AD	HEK293 cells	-	Increased membrane fluidity and non-amyloidogenic processing of APP, leading to enhanced secretion of sAPPα. This enhanced secretion of sAPPαwas associated with substantial protection against apoptosis induced by ER Ca2+store depletion	[40]
Flavones	Oxidative stress	ND	SH-SY5Y	-	Neuroprotection was found to be mediated via activation of the anti-apoptotic cell survival proteins of the ERK1/2 and PI3K/Akt pathways. S	[41]

Erythropoietin (hormone)	BDNF and other neurotrophins	ND	*in vitro* and *in vivo*	rats (brain)	Prevention of HAL-induced reduction in BDNF, increase in caspase-3 and decrease in Bcl-xl protein levels.	[42]
17β-Estradiol (steroid)	Not fully established	RD	Primary rat RNCs	rats (brain)	Suppression the Bax-involved mitochondrial apoptosis to protect the RNCs from H_2O_2 insult by activating the PI3K/Akt pathway	[43]
JM-20 (anxiolytic)	Mitochondria	ND	-	rats (brain)	Strong antioxidant action and protective effects against Ca2+-induced impairment, which are both elicited at the mitochondrial level	[44]
Leupeptin, calpain inhibitor XI AND Z-VAD.FMK	Calpains and caspases	ALS	-	mice (spinal cord)	Leupeptin and calpain inhibitor XI inhibited apoptosis in the motor neurons while Z-VAD.FMK had no effect. Leupeptin, but not calpain inhibitor XI and Z-VAD.FMK inhibited nucleosomal DNA fragmentation	[45]
Lixisenatide/liraglutide (GLP-1)	GLP-1 receptor	PD	-	mice (brain)	Exendin-4 showed no protective effects at the dose chosen. Both liraglutide and lixisenatide prevented the MPTP-induced motor impairment, reduced dopamine synthesis; and promoted decrease in Bax and an increase in bcl-2	[46]
LMWSC (sulfated chitosan is the structural analog of heparin converted to low molecular weight polymer by γ-irradiation)	Lipids	PD	SH-SY5Y	-	Reduction of the intracellular ROS levels, normalization of antioxidant enzymes, mitigation of rotenone induced mitochondrial dysfunction and apoptosis	[47]
Melatonin	Mitochondria	ND	-	mice (brain)	Protective role against H-89-induced memory impairment	[48]
Memantine	N-methyl-D-aspartate receptor antagonist	AD	SH-SY5Y	-	Upregulation of neuronal cell survival, inhibition of neuronal autophagy and apoptosis. Upregulation of the expression of signaling molecules phosphorylated (p)-phosphoinositide 3-kinase, p-Akt and p-mammalian target of rapamycin (mTOR), and inhibition the	[28]

					phosphorylation of bcl2/Beclin-1 complex via mitogen-activated protein kinase 8.	
Minocycline	Microglia	PD	Primary neuronal culture	mice (brain)	Inhibition of microglial activation by minocycline protects against the rotenone toxic effects and prevented the dropout of tyrosine hydroxylase and apoptosis by rotenone	[49]
N-acetyl-l-tryptophan (L-NAT)		ALS	NSC-34 motor neurons	mice (brain)	Neuroprotective in primary motor neurons by inhibition of the secretion of Substance P and IL-1β and mitochondrial dysfunction by inhibiting the release of cytochrome c/Smac/AIF and activation of apoptotic pathways (caspase-1, -9, and -3), as well as proteasomal dysfunction through restoring chymotrypsin-like, trypsin-like, and caspase-like proteasome activity.	[50]
Nicotinamide	Mitochondria	ND	-	rats (brain)	Inhibition of ketamine-induced neuro-apoptosis by downregulating Bax, inhibiting cytochrome c release from mitochondria into cytosol, and inhibiting the expression of activated caspase-3	[51]
Olomoucine/BAF	Cyclin-dependent kinase/caspases	AD	SH-SY5Y	-	Cyclin-dependent kinase inhibitor reduced camptothecin-induced cell death and decreased the effects on tau phosphorylation. The general caspase inhibitor decreased camptothecin-induced cell death, but did not significantly affect tau phosphorylation.	[52]
Oxyntomodulin analog (hormone and growth factor)	GLP-1 and the glucagon receptor.	PD	-	mice (brain)	Prevention of the MPTP-induced motor impairment, reduction in TH levels (dopamine synthesis), the reduction of the synaptic marker synaptophysin, the inactivation of the growth factor kinase Akt/PKB and of Bcl-2, and the increase pro-inflammatory cytokine TNF-α levels	[53]

P7C3 (aminopropyl carbazole)	Mitochondria	PD	Dopaminergic cell line	mice (brain)	P7C3 stabilized mitochondrial membrane potential, reduced reactive oxygen species production, and inhibited GSK3β activation, p53 activity, Bax upregulation and cytochrome c release exposed to MPP+, and prevented neuronal loss in the substantia nigra	[54, p. 3]
Peroxiredoxin	Oxidative stress and apoptosis (via signal-regulating kinase (ASK1)-dependent activation of the c-Jun N-terminal kinase/c-Jun and p38 pro-death pathways)	PD	MN9D neuronal cells and human brains	mice/human (brain)	*In vitro* and *in vivo* neuroprotection against 6-OHDA toxicity in DA neurons, and preserved motor functions involving the dopamine system in mouse. PRX2 exhibited antioxidant and anti-apoptotic effects via suppression of apoptosis signal-regulating kinase (ASK1)-dependent activation of the c-Jun N-terminal kinase/c-Jun and p38 pro-death pathways	[55]
Pridopidine	Dopamine stabilizer	PNS	-	mice (brain)	Increased expression of pro-survival and neurostimulatory molecules BDNF and DARPP32, reduction in the size of mHtt aggregates in striatal tissues	[56]
Quercetin (flavonoid)	Antioxidant/anti-inflammatory	ND	-	rats (brain)	Quercetin supplementation decreased the neuronal damage and scavenged the free radicals induced by PCBs and protects PCBs induced apoptosis and oxidative stress.	[57]
Retinoic acid	Proteasome	PD	SH-SY5Y cells	human (neuroblastoma)	A treatment of cultured neuroblastoma cells sets up conditions under which proteasome inhibition, and the resultant accumulation of ubiquitinated proteins, loses its ability to kill the cells	[58]
Sildenafil (phosphodiesterase type 5 inhibitor)	Cyclic GMP phosphodiesterase	MS	-	mice (spinal cord)	Inhibited nitrosative stress and augmented the levels of LC3, beclin-1, ATG5, p-CREB and BDNF and decreased mTOR levels, as well as augmented p-AMPK.	[59]
Triple GLP-1/GIP/glucagon	GIP-1, GIP and glucagon receptors	AD	-	mice (brain)	Reversion the memory deficit in the APP/PS1 mice	[60]

receptor agonist (triple agonist)					in a spatial water maze test and reduction of the levels of Bax, increased Bcl-2, BDNF and synaptophysin, neurogenesis enhancement in the dentate gyrus, decrease of β-amyloid, neuroinflammation and oxidative stress	
Voltage channels (agonist for the AMPA receptor where it mimics the effects of the glutamate)	AMPA receptor Reduced caspase-3 activity	SZ	-	rats (brain)	Pretreatment with the calcium channel blocker cadmium chloride eliminated AMPA-mediated protection against PCP. VDCCs might be advantageous in treating conditions associated with diminished NMDAergic activity during early development.	[61]
VDAC1-derived peptide	VDAC (mitochondria)	AD	SH-SY5Y and PC12	-	Aβ Entry into SH-SY5Y Cells Is Inhibited by the VDAC1 N-Ter Peptide	[62]
Y27632/NAD+/ZVAD-FMK/resveratrol	Kinase/Caspase	ND	Primary neuronal culture	mice (brain)	Y27632 and NAD+ exert strong synapto-protective activities whereas zVAD-FMK and resveratrol fail to protect synapses	[63]
Zileuton	5-Lipoxygenase (5-LOX)	ND	HT22 mouse neuronal cells	mice (brain)	Neuroprotection from glutamate-induced oxidative stress at least in part by inhibiting ferroptosis	[64]
ZVAD-FMK/IETD-FMK	Caspase	PD	SH-SY5Y	-	Complete apoptotic prevention by the broad caspase inhibitor zVAD-FMK but not by the caspase-8 inhibitor IETD-FMK.	[65]
Z-VAD-FMK/Z-DEVD-FMK/Z-LEHD-FMK	Caspase	ND	-	sparrow (brain)	Reduction of programmed cell death by local infusion of caspase inhibitors rescues a telencephalic nucleus in the adult avian song control system from hormone withdrawal-induced neurodegeneration	[66]

6-OHDA, 6- hydroxide dopamine; AD, Alzheimer's disease; ALS, amyotrophic lateral sclerosis; AMPA, α-amino-3-hydroxy-5-methyl-4-isoxazoleproprionic acid; APP, β-amyloid precursor protein; BAF, Boc-aspartyl (OMe)-fluoromethylketone; BDNF, brain derived neurotrophic factor; Ct, clinical trial; DHA, omega-3 fatty acid docosahexaenoic acid; GIP, glucose-dependent insulinotropic polypeptide; GLP-1, Glucagon-like peptide 1; HAL, haloperidol; HD, Huntington's disease; hiNPS, human induced neuroprogenitor stem cells; HPA, hypothalamic–pituitary–adrenal; GSK3β, glycogen synthase kinase-3 beta; L-NAT, N-acetyl-l-tryptophan; LMWSC, low molecular weight sulfated chitosan; MPP+, 1-methyl-4-phenylpyridinium; MS, multiple sclerosis; NAD+ nicotinamide adenine dinucleotide; ND, neurodegeneration; PD, Parkinson's disease; PNS, prenatal stress; RD, retinal degeneration; RNCs, retinal cells; ROS, reactive oxygen species; TH, tyrosine hydroxylase; Y27632, Rho Kinase inhibitor * This study based on prenatal stress induction in rats represents a close model with homology to SZ models; SZ, schizophrenia.

Supplementary Table S6. Quantitative data of molecular and clinical outcomes by using anti-apoptotic compounds in models of neurodegeneration and neuroprogression.

Compound	Target/interaction	Disease	Model	Source of sample	Parameter	Outcome	Treated group	Control group	Sample size	P value	Reference
Albumin	Mitochondria	ND	ER stress induced SH-SY5Y	SH-SY5Y neuroblastoma	Apoptosis	Apoptotic rate induced by flunarizine (% cells)	14%	65%	n=15	p<0.05	[67]
					Apoptosis	Apoptotic rate induced by cyclopiazonic acid (% cells)	23%	8%	n=15	p<0.05	
Ac-LETD-CHO	Caspase-3	ND	glucotoxicity-induced neuropathy	ND7/23 neuronal cells	Apoptosis	Caspase-3	35%$^{\vee}$	-	n=15	p=0.02	[68]
Anthocyanins	AMPK pathway	ND		Mouse hippocampal cells (HT22)	% cells with low ΔΨmt	$^{\vee}$ΔΨmt	35.56±8.5%	65±4.1%	n=12	p<0.05	[69]
Asiatic acid	Oxidative stress, mitochondria, inflammation	PD	Rotenone induction	SH-SY5Y	Cell survival	MTT (%)	55.37%^		n=28	p<0.05	[70]
Asiaticoside	Unclear anti-apoptotic mechanism	AD	Aβ1-42 induced	human brain microvascular endothelial cells	Apoptosis	Annexin V+/PI+ (apoptosis %)	35.20 ± 5.26%	66.40 ± 4.82%	n=18	p<0.01	[71]
BAF	Caspases	MSA	NGF-withdrawal	superior cervical ganglia neurons	Neuronal resistance	Neuronal action potential (MΩ)	577±45	198±21	n=36	p<0.05	[72]
Baicalein	Proteasome	PD		NGF-differentiated PC12 cells	Cell death	MTT	51%$^{\vee}$	-	n=24		[73]
bpV(pic)	Phosphatase and tensin homolog (PTEN)	AD	Aβ 25-35	SH-SY5Y	Apoptotic rate	Annexin V+	12.8±2.2%	25.2±4.6%	n=18	p<0.05	[74]
B355252	Nerve growth factor	PD	6-OHDA induced	HT22	Cell viability	Resazurin (7-Hydroxy-3H-phenoxazine-3-one 10-oxide)	31.7 %^	76.6 %$^{\vee}$	n=15	p<0.001	[75]
						JNK1/2 phosphorylation (relative units)	16.8 %$^{\vee}$	65%^	n=15	p<0.001	
C. tomentosum extract					Cell viability	MTT (% of control)	81.29 ± 3.68%	56.41±5.05	n=21	p<0.05	[76]
					Mitochondrial membrane potential	Monomers/aggregates JC1 (%)	128.00±5.61%	191.17±6.52%	n=21	p<0.05	

CGP 3466B	Glyceraldehyde-3-phosphate dehydrogenase	ALS			Disease progression	Disease progression (days)	30%$^{\vee}$	-		p<0.05	[77]
					Lifespan	Lifespan (days)	53.7±2.5 %	42.3±0.8	n=30	p<0.05	
						Motor neurons (counts)	733±41	548±46	n=13	p<0.05	
CHA79*	Oxidative stress, neurotrophic factors, glyoxalase	ND	MG-mediated neurotoxicity	SH-SY5Y cells	Cell viability	MTT (%)	78.59±3.68%	60.61±2.70%	n=30	p<0.05	[78]
					Apoptosis	Annexin V+ (%)	12.49±0.82%	21.46±1.78%	n=30	p<0.05	
					Oxidative stress	ROS production H2DCF-DA staining (DCF fluorescence %)	121.54 ± 7.75%	142.80 ± 5.08	n=30	p<0.05	
					Oxidative stress (antioxidant enzymes)	SOD activity (U/mg protein)	39.38 ± 2.49	25.34 ± 1.69	n=30	p<0.05	
					Oxidative stress (antioxidant enzymes)	GSH levels (OD 405 nm, nmol/mg protein)	52.90 ± 1.25	38.84 ± 1.89	n=30	p<0.05	
Cycloheximide	Protein synthesis	ND	MK801 induced	rat retrosplenial cortex	Neuronal death	Nº of dying neurons	33±18	510 ±20	n=28	p<0<001	[79]
Dapagliflozin	Sodium-glucose co-transporter-2	HD	3-NP exposure		Behavioral data (beam)	Time to cross the beam	32%$^{\vee}$	-	n=40	p<0.01	[80]
					Behavioral data (retraction time)	Time (retraction time)	61%$^{\vee}$	-	n=40	p<0.01	
					Behavioral data (transfer latency)	Time (transfer latency)	56%$^{\vee}$	-	n=40	p<0.01	
FK506	Calcineurin	HD	3-NP exposure	Primary mice striatal neurons	Cell death necrosis vs. Controls	Cell death (% total cells)	10.77±2.39%$^{\vee}$	-	n=15	p<0.05	[81]
Forsythiaside	Oxidative stress, bacteria, inflammation	AD	H2O2-induced toxicity	PC12 cells	Apoptosis	% Annexin V+ cells	8.49 %	24.52 %	n=12	p<0.01	[82]
Fucoidan	Mitochondria	AD	Aβ25–35 and D-Gal-induced neurotoxicity		Behavioral data (water maze)	Escape latency time (s)	28.52±16.51	38.62±19.51	n=24	p<0.05	[83]

					Oxidative stress	Cell ROS deep red 640/655 nm	41.5 %v	50 %^	n=15	p<0.001	
Gallic acid (Corni fructus)	Oxidative stress				Oxidative stress	ROS production CM-H2DCFDA (DCF fluorescence)	73.00 ± 13.05%	100 ± 3.83%	n=12	p< 0.001	
Hepad 1 and 2	Caspase-3/9	PD	MPTP	C57BL/6 mice and SH-SY5Y cells	Cell viability	MTT (%)	86.5%^	-	n=18	p<0.05	[84]
					Oxidative stress	NOX-4 protein levels (intensity)	1.43xv	-	n=18	p<0.05	
					Cell cycle (extracellular signal regulated kinases)	p-ERK protein levels (intensity)	1.1xv	-	n=18	p<0.05	
					Dopaminergic viability	TH+ neurons	2x^	-	n=18	p<0.05	
					Inflammatory response	TNFa protein levels	2.7xv	-	n=18	p<0.05	
					Inflammatory response	IL6 protein levels	1.5xv	-	n=18	p<0.05	
					Inflammatory response	iNOS protein levels	2.9xv	-	n=18	p<0.05	
					Inflammatory response	Mac-1 protein levels	1.2xv	-	n=18	p<0.05	
					Apoptosis	p53 protein levels (intensity)	1.4xv	-	n=18	p<0.05	
					Apoptosis	Bax protein levels (intensity)	1xv	-	n=18	p<0.05	
					Anti-apoptotic protein family	Bcl-2 protein levels (intensity)	1.2x^	-	n=18	p<0.05	
					Apoptosis (activation signaling)	PARP1 protein levels (intensity)	1.1xv	-	n=18	p<0.05	
					Oxidative stress (antioxidant enzymes)	SOD protein levels (intensity)	2.2x^	-	n=18	p<0.05	
					Oxidative stress (antioxidant enzymes)	GST protein levels (intensity)	2.9x^	-	n=18	p<0.05	
					Cell survival signaling regulation	p-AKT	1xv	-	n=18	p<0.05	
iMACs	Mitochondria	ND	Staurosporine	Mitochondria from FL5.12 cells	Onset MOMP	Onset MOMP* (h)	12.52±0.9 h	6.83±1.1h	n=21	p<0.001	[85]

					Apoptosis	Bax fluorescence (%)	22%	81%	n=22	p<0.001	
JJDHYZ	Mitochondria and ER	PD	MPTP-induced	Corpus striatum	Monoamine neurotransmitters	Dopamine (pg/ul)	174.1±34.9	54.8 ± 15.4	n=25	p<0.01	[86]
JM-20	Oxidative stress, excitotoxicity, inflammation	AD	Scopolamine		Antioxidant activities	SOD (U/min mg protein)	4.31±0.044	2.89±0.003	n=50	p<0.05	[87]
					Antioxidant activities	CAT (U/min mg protein)	8.21±1.874	4.77±0.349	n=50	p<0.05	
					Total tissue SH	TSH (umol/mg protein)	1.99±0.167	1.23±0.134	n=50	p<0.05	
					Lipid peroxidation	MDA (nmol/mg protein)	2.01±0.230	3.84±0.490			
Licochalcone A	JNK1	ND	Kainic acid induced	Hippocampus	Apoptotic cascade biomarkers	BCL-2 (AU)	14%^	-	n=60	p<0.01	[88]
					Apoptotic cascade biomarkers	Bax	31%˅	-	n=60	p<0.01	
					Apoptotic cascade biomarkers	Bim	48.9%˅	-	n=60	p<0.01	
					Apoptotic cascade biomarkers	CaSBC	38.7%˅	-	n=60	p<0.01	
					Apoptotic cascade biomarkers	CaSBC3	49.1%˅	-	n=60	p<0.01	
					Disease outcomes	Seizures	1.83±0.17	2.46±0.16	n=30	p<0.05	
					Survival pathways	Phosphorylated AKT	45.6%˅	-	n=60	p<0.01	
					Mitochondrial stress responses	PGC1a	9.5%˅	-	n=60	p<0.01	
					Neuroinflammatory responses	TNFa	33.5%˅	-	n=60	p<0.01	
					Neuroinflammatory responses	Induced nitric oxide synthase	38.49%˅	-	n=60	p<0.01	
					Neuroinflammatory responses	Neuronal nitric oxide synthase	25.8%˅	-	n=60	p<0.01	
					Metalloproteases associated activation of soluble TNFa	ProADAM10	40.31%˅	-	n=60	p<0.01	
					Metalloproteases	ADAM10	24.8%˅	-	n=60	p<0.01	

					associated activation of soluble TNFa						
MADP (salidroside analog)	Oxidative stress, inflammation	ND	Glutamate induced	Culture hippocampal neurons	Cell viability	MTT	75.5%^	60.9%ᵛ	n=18	p<0.05	[89]
					Apoptotic rate	Apoptotic cell counts (%)	59.2%	73.6%	n=18	p<0.01	
					Apoptotic rate	Annexin V+	30.2%	55.1%	n=18	p<0.01	
					Necrosis	Annexin V+/PI+	1.7%	8.2%	n=18	p<0.01	
Methylene blue	Caspase-6	AD	Caspase-6 overexpressing	Hippocampus (mice)	Synaptic function (theta-burst LTP*)	fEPSP slope	141.22%	132.42%	n=42	p=0.0003	[90]
Metformin	Mitochondria	ND	Etoposide-induced	Primary cortical neurons (rat)	Calcium retention capacity (PTP)	Nmol Ca2+/10e7 cells	50%^	-	n=12	p<0.05	[91]
Methylene blue	Mitochondria	ON	Rotenone induction	Retinal ganglion cells (Inner plexiform layer)	Cytochrome oxidase activity	Cytochrome oxidase activity	22%^	-	n=18	p=0.036	[92]
					Oxidative stress	TEP U/ml	1.1 ± 0.05	4.6 ± 0.1	n=9	p =0.004	
					Cell viability	Calcein-Acetomethoxy/ Ethidium Homodimer-1	1.6-fold ^		n=6	p< 0.001	
					Behavioral data (water maze)	Water maze (correct choices)	83.5±8%	49.3 ± 2%	n=28	p = 0.298	[93]
M17Z protein	Caspase-1	HD	Transgenic R6/2 mice		Disease onset	Days of disease onset	84.4±2.0	77.1±1.4	n=21	0.012	[17]
M40403*	Oxidative stress	SZ	Phencyclidine induced	Striatum	Apoptosis	TUNEL+ cells	6.3±1.3	9.0±1.9	n=24	p<0.05	[94]
					Disease length	Days of disease length	36.8±3.3	23.8±2.4	n=21	0.0075	
					Mortality	Mortality (days)	121.3±3.7	100.9±2.5	n=21	0.0007	
Minocycline	Caspases, mitochondria, microglia	HD	Human patients	Human patients	Motor function	Chorea	7.0±7.1	8.9±6.3	n=11	p=0.067	[95]
					Neuropsychological function	Symbol digit modalities test	26.0±8.9	35.3±9.3	n=11	p=0.008	
					Psychiatric symptoms	Total psychiatric score	3.8±5.5	13.2±8.5	n=11	p=0.015	
					Psychiatric symptoms	Depressive symptoms	2.4±3.4	9.1±6.0	n=11	p=0.016	
					Psychiatric symptoms	Apathy	0.9±1.8	2.7±1.8	n=11	p=0.005	

		D/SZ			Depression	HAM-D-21	6.7±1.9	40.4 ± 2.5	n=25	p<0.05	[96]
					Psychotic symptoms	BPRS scores	4.6±2.4	63.3 ± 8.7	n=25	p<0.05	
		AD			Mitochondrial respiration	State 2	38.3±3.3	21.88±3.8	n=12	p<0.05	[97]
					Mitochondrial respiration	State 3	91.5±13.5	150.7±24.1	n=12	p<0.05	
					Mitochondrial respiration	State 4	47.8±5.8	22.0±2.8	n=12	p<0.05	
					Mitochondrial respiration	Respiratory control ratio	1.95±0.48	6.83±0.24	n=12	p<0.05	
Morinda citrifolia	Oxidative stress	PD	Rotenone induced	Striatum	Mitochondrial respiratory chain	Complex I	22%^	-	n=50	p<0.05	[98]
						Complex IV	23%^	-	n=50	p<0.05	
					Intrinsic apoptosis	Cytochrome c release	12%	28%	n=50	p<0.05	
Naringin	Oxidative stress	HD	3-NP	striatum	Antioxidant system	SOD	10.84±1.02	6.8±0.57	n=24	p<0.05	[99]
					Antioxidant system	Catalase	1.92±0.15	1.42±0.14	n=24	p<0.05	
					Antioxidant system	GPx	14.38±1.21	9.88±0.78	n=24	p<0.05	
					Antioxidant system	GR	2.22±0.17	1.42±0.12	n=24	p<0.05	
					Lipid peroxidation	Nmoles MDA/mg protein	1.6±0.15	1.03±0.09	n=24	p<0.05	
					Protein carbonyl levels	Protein carbonyl-nmol/mg protein	4.32±0.29	6.45±0.45	n=24	p<0.05	
					Membrane bound ATPases	Na+/K+- ATPase	0.33±0.03	0.69±0.06	n=24	p<0.05	
					Membrane bound ATPases	Ca2+-ATPase	0.23±0.02	0.32±0.03	n=24	p<0.05	
					Membrane bound ATPases	Mg2+/ATPase	0.30±0.03	0.46±0.04	n=24	p<0.05	
Osthole	Mitochondria	PD	MPP+-induced cytotoxicity	PC12 cells	Cell viability	MTT	32%^	-	n=15	p<0.01	[100]
					Cell toxicity	LDH release	160.8 ± 4.6%	210.9±5.8%	n=15	p<0.01	
					Morphology of cell nuclei	Nuclear condensation	40.2±5.9%	48.9±6.4%	n=15	p<0.05	
					Apoptotic rate	Annexin V/PI	35.8%	59.4%	n=15	p<0.05	
					Intracellular ROS	DCFH-DA fluorescent probe 485/535nm (%)	152.5±7.9 %^{v}	-	n=15	p<0.01	

					Mitochondrial membrane potential collapse	Fluorescence intensity (%)	51.6±4.6 %	43.9±4.8%	n=15	p<0.01	
					Apoptotic rate	Bax/ Bcl-2 ratio	22.3%˅	2.1x^	n=15	p<0.01	
					Apoptotic rate	Cytochrome c release (AU)	27.5%˅	-	n=15	p<0.01	
Oxotremorine-M	Muscarinic receptors	ND	camptothecin-induced	SH-SY5Y	Apoptosis	Caspase-3 activity	75%˅		n=16	p<0.05	[101]
Paeoniflorin	Oxidative stress and mitochondria (not yet fully delineated)	PD	6-OHDA induced	PC12 cells	DNA fragmentation	Cytoplasmic histone-associated-DNA-fragments	39%˅	-	n=15	p<0.05	[102]
					Oxidative stress	(DCFH-DA) 485/538nm	9%˅	-	n=15	p<0.05	
					Apoptosis	% Annexin V+ cells	16%˅	-	n=15	p<0.05	
					Antioxidant system	GSH content (nmol/mg protein)	28%^	-	n=15	p<0.05	
		ND		differentiated PC12 cells	Cell viability	MTT (%)	7%	58%	n=9	p<0.05	[103]
						LDH release (%)	87%	143%	n=9	p<0.01	
					Apoptosis	Annexin V/PI (Q2+Q3)	6.1%	16.8%	n=9	p<0.01	
					Calcium overload	Calcium levels (%)	112%	144%	n=9	p<0.05	
					Apoptosis	Cleaved PARP (intensity, %)	55%	60%	n=9	p<0.05	
					Anti-Apoptotic protein family	Bcl-xL (intensity, % of controls)	94%	21%	n=9	p<0.05	
PF9601N*	MAO-B	PD	MPTP	SH-SY5Y cells	Cell viability	MTT (%)	69.5 ± 2.3%	24.2 ± 1.5%	n=27	p<0.05	[104]
P-coumaric acid (Corni fructus)	Apoptosis	AD	Aβ(25–35)	PC12 cells	Cell viability	MTT (%)	98.89 ± 7.61%	66.55 ± 2.84%	n=12	p< 0.001	[105]
					Oxidative stress	ROS production CM-H2DCFDA (DCF fluorescence)	68.86 ± 9.69%,	100 ± 3.83%	n=12	p< 0.001	
P. pavonica extract	Oxidative stress				Oxidative stress	H_2O_2 production	208.74±5.09%	363.81±28.58%	n=21	p<0.05	
Q-VD-OPH	Caspases	PD, HD	MPTP, NP3 and malonate induced	Striatum	Neurotransmitter levels	Striatal dopamine (ng/ml protein)	200%^	30%˅	n=14	p<0.05	[106]
		VE	Reovirus infected mice	Brain slice cultures	Viral induced injury	Viral induced injury	35%˅	-	n=8	p=0.008	[107]

RBC1023	Caspase-3	ND	Staurosporine	PC12 rat pheochromocytoma	Cell viability	MTT*	66%	4%	n=18	p<0.01	[108]
	Caspase-3	ND		U87MG human glioblastoma		MTT	71%	8%	n=18	p<0.01	
	Caspase-3	ND		SH-SY5Y neuroblastoma cells		MTT	47%	6%	n=18	p<0.01	
Salidroside						MTT	67.1%^	60.9%ᵛ	n=18	p<0.05	[89]
						LDH release	34.3%ᵛ	40.6%^	n=18	p<0.05	
					Apoptotic rate	Apoptotic cell counts (%)	64.2%	73.6%	n=18	p<0.01	
						Annexin V+/PI+	38.1%	55.1%	n=18	p<0.05	
						MTT	5.2%	8.2%	n=18	p<0.05	
S. muticum extract	Oxidative stress	PD	Dopamine induced	SH-SY5Y cells	Cell viability	MTT (% of control)	82.37±6.41%	56.41±5.05	n=21	p<0.05	[76]
					Mitochondrial membrane potential	monomers/aggregates JC1 (%)	135.70±2.97%	191.17±6.52%	n=21	p<0.05	
					Apoptosis	Caspase-3 activity (fluorescence a.u.)/mg of protein/min)	2.53±2.49	66.46±1.49	n=21	p<0.05	
S. polyschides extract	Oxidative stress				Cell viability	MTT (%)	89.26±8.62%	56.41±5.05	n=21	p<0.05	[76]
					Apoptosis	Caspase-3 activity (fluorescence a.u./mg of protein/min)	4.71±0.70	66.46±1.49	n=21	p<0.05	
					ATP levels	ATP levels (%)	1.2x^	-	n=24	p<0.01	
					MMP	ΔΨmt (JC1, %)	80%	50%	n=24	p<0.001	
TRP601*	Caspases 2/3		Oxygen induced	thalamic tissue	Apoptosis	Caspase-3 activity	1,28	1,86	n=18	p<0.01	[109]
						Caspase-2 activity	1,58	358%	n=24	p<0.01	
Trifluoperazine/ W-7	Calmodulin	ND	MG132-induced	PC12 cells	Cell death	MTT (death counts)	47%ᵛ	55%^	n=16	p<0.05	[110]
Tropisetron	Serotonergic system	AD	β-amyloid (Aβ) rat model	Hippocampus	Proinflammatory cytokine	TNFα (pg/mg protein)	59.428±8.2	148.773±10.3	n=76	p<0.001	[111]
U. compressa extract	Oxidative stress				Mitochondrial membrane potential	Monomers/aggregates JC1 (%)	126.73±5.46%	191.17±6.52%	n=21	p<0.05	[76]
Ursolic acid (Corni fructus)	Oxidative stress				Oxidative stress	ROS production CM-H2DCFDA (DCF fluorescence)	51.69 ± 2.88%	100 ± 3.83%	n=12	p< 0.001	
Vanillyl alcohol (Gastrodia elata)	Oxidative stress, apoptosis	PD	MPTP	MN9D dopaminergic cells	Cell viability	MTT (%)	76.3 ± 4.6%	50.5 ± 1.4%	n=18	p<0.05	[112]

					Apoptosis	PI (%)	16.8±2.8	37.6 ± 1.2%	n=18	p<0.05	
					Cell death signaling	PARP proteolysis	143.24 ± 7.97%	251.14 ± 17.39%	n=18	p<0.05	
XIAP and p35	Caspases	ALS	SOD1 transgenic mice		Mean survival	Survival (days)	276.6±64.8	258.96±4.1	n=13	p<0.001	[113]
					Mean disease duration	Death-onset (days)	34.6±2.6	22.6 ±1.7	n=13	p<0.0005	
					Surviving motor neurons	Nº motor neurons ratio	67.5±3.7%	53.2±2.5%	n=13	p<0.01	
Z-VAD-FMK	Caspases and calpains	ALS	Transgenic mice	Mice	Time before disease onset	Days before disease onset	123.76±6.8	103.56±2.8	n=19	p=0.048	[19]
					Time of survival	Days of survival	153.36±8.8	126.16±3.0	n=19	p=0.011	
		HD	Transgenic mice	R2/6 mice	Time of survival	Days of survival	98.6	79	n=5	p=0.002	[17]
				Substantia nigra pars compacta	Neuronal amount	TH+ dopaminergic neurons	17%^	38%ᵛ	n=14		
		HD	3NP-induced	Striatal cells	Cytochrome c oxidase	COX levels (OD units)	25.6±7.1%	17.7±3.5%	n=19	p=0.0007	[114]
		ON	Inherited retinal degeneration	rat eyes	Electroretinography	Maximal b-wave amplitude (µV)	511±92	331±58	n=34	p = 0.0003	[115]
					Apoptosis	Nº apoptotic nuclei (inferior hemisphere)	63%ᵛ	37.4±15.2	n=8	p<0.05	
						Nº apoptotic nuclei (superior hemisphere)	60%ᵛ	35.3±10.9	n=8	p<0.05	
		PD	6-OHDA	Substantia nigra	Nigral dopaminergic neurons	TH+ counts	0.6	0.2		p<0.01	[116]
11-dehydrosinulariolide	Inflammation	PD	6-OHDA induced	SH-SY5Y cells	Cell viability	Relative protection (%)	15.8±7.3	0.0±3.5	n=50	p<0.05	[117]
					Nuclear translocation rate	NF-κB localized to the nucleus (% cells)	22.5±5.2%	48.0±8.1%	n=50	p<0.05	

3-NP, 3-Nitropropionic acid; 6-OHDA, 6-hydroxydopamine; Aβ 25-35, amyloid beta peptides; AD, Alzheimer's disease; ALS, amyotrophic lateral sclerosis; ATP, adenosine triphosphate, B355252, 4-chloro-N-(naphthalen-1-ylmethyl)-5-(3-(piperazin-1-yl)phenoxy)thiophene-2-sulfonamide); BAF, boc-aspartyl(OMe)fluoromethyl ketone; CAT, catalase; CGP 3466B, dibenzo[b,f]oxepin-10-ylmethyl-methyl-prop-2-ynyl-amine; CHA79, 2-iodo-40 -methoxychalcone; D, depression; GSH, glutathione; GST, glutathione transferase; Huntington's disease; iMACs, Mitochondrial Apoptotic Channel Inhibitors; JJDHYZ, Jia-Jian-Di-Huang-Yin-Zi decoction; LTP, long term potentiation; M40403, superoxide dismutase mimetic; MDA, malondialdehyde; MMP, mitochondrial membrane potential; MOMP, mitochondrial outer membrane permeabilization; MPTP, 1-methyl-4-phenyl-1,2,3,6-tetrahydropyridine; MSA, multiple system atrophy; MTT, 3-(4,5-dimethylthiazol-2-yl)-2,5-diphenyltetrazolium bromide; N-benzyloxycarbonyl-valyl-alanyl-aspartyl-fluoromethylketone; ND, neurodegeneration; OD, Optical density; ON, optical neuropathy; PD, Parkinson's disease; PF9601N, [N-(2-propynyl)-2-(5-benzyloxy-indolyl) methylamine]; PI, propidium iodide; ROS, reactive oxygen species; SOD, superoxide dismutase; SZ, schizophrenia; TH, tyrosine hydroxylase; TRP601, 5-(2,6-difluoro-phenoxy)-3(R,S)-(2(S)-(2(S)-(3-methoxycarbonyl-2(S)-(3-

methyl-2(S)-((quinoline-2-carbonyl)-amino)-butyrylamino)propionylamino)3-methylbutyrylamino)propionylamino)-4-oxo-pentanoic acid methyl ester; VE, viral encephalitis; ZVAD, Z-Val-Ala-Asp-CH2F.

Supplementary Table S7. Therapeutic approaches against SZ with compounds indirectly targeting apoptosis.

Substance	Action/interaction	Conclusions	References
DHA, omega-3 fatty acids	Antioxidant	Current data not conclusive and do not allow either to refuse or support the neuroprotective role of omega-3 fatty acids in SZ	[118]
Erythropoietin	BDNF and other neurotrophins	Effective in treating cognitive deficits associated with SZ	[119]
Estradiol	Not fully established	Improvements in primary outcomes of childbearing age women	[120]
Flavones	Antioxidant	Quercetin successfully augmented antipsychotic effects in treatment-resistant SZ. Hispidulin and morine attenuate SZ-like behaviors in SZ in animal models	[28], [121], [122]
Melatonin	Antioxidant	Clinical trial showed effective treatment for SZ sleep disorders, Tardive dyskinesia and Benzodiazepines discontinuation	[123]–[125]
Memantine	N-methyl-D-aspartate receptor antagonist	Improvement of the verbal memory, learning, verbal letter fluency, and working memory without improvement on psychotic symptoms	[126]
Minocycline (antibiotic)	Caspases	Amelioration of cognitive deficits that correlated with remission of negative symptoms, and reduction in serum levels of IL-1β and IL-6	[127]
N-acetylcysteine	Antioxidant	Improvement in positive, negative, general and total psychopathology symptoms as well as cognitive performance. Antiapoptotic effects derived from NAC have been previously reported	[128], [129]
Nicotinamide and nicotinic acid	Mitochondria	No evidence to support the hypothesis that either nicotinic acid or nicotinamide is useful as adjuvant therapy in the treatment	[130]
Quercetin (flavone)	Antioxidant/anti-inflammatory	2 case reports suggest effectiveness to augment antipsychotic treatment	[121]
Retinoic acid (bexarotene)	Proteasome	Clinical trials with an add-on retinoid agonist (bexarotene) showed improvement of positive symptoms in SZ patients.	[131]
Sildenafil	Phosphodiesterase type 5 inhibitor	Despite evidence for cognitive-enhancing effects of sildenafil in animal models, the strategy	[132]

		for treating putative NMDA receptor-mediated memory deficits and depression in SZ was not successful. It is possible that the doses used in this study were not optimal or that repeated dosing may be necessary to achieve therapeutic effects.	
Vitamin C	Antioxidant	Case reports, double-blind randomized clinical and open trials (46) indicate that the symptoms of chronic SZ can be ameliorated by high-dose ascorbic acid therapy	[133]–[135]

References

1. Zhu, L.-F.; Wu, X.-Y.; Liu, X.; Ma, L.; Zhang, Q.; Yu, Z.; He, Z.-J.; Ying, Y.; Zhang, Z.-T.; Pan, X.-Y.; et al. Absence of BAX differentially affects astrocyte density in the mouse cortex and hippocampus. *Sheng Li Xue Bao Acta Physiol. Sin.* **2021**, *73*, 1–9.
2. D'Orsi, B.; Kilbride, S.M.; Chen, G.; Alvarez, S.P.; Bonner, H.P.; Pfeiffer, S.; Plesnila, N.; Engel, T.; Henshall, D.C.; Düssmann, H.; et al. Bax Regulates Neuronal Ca2+ Homeostasis. *J. Neurosci.* **2015**, *35*, 1706–1722. https://doi.org/10.1523/jneurosci.2453-14.2015.
3. Smith, M.I.; Deshmukh, M. Endoplasmic reticulum stress-induced apoptosis requires bax for commitment and Apaf-1 for execution in primary neurons. *Cell Death Differ.* **2007**, *14*, 1011–1019. https://doi.org/10.1038/sj.cdd.4402089.
4. Wyttenbach, A.; Tolkovsky, A.M. The BH3-only protein Puma is both necessary and sufficient for neuronal apoptosis induced by DNA damage in sympathetic neurons. *J. Neurochem.* **2006**, *96*, 1213–1226. https://doi.org/10.1111/j.1471-4159.2005.03676.x.
5. Vila, M., Jackson-Lewis, V., Vukosavic, S., Djaldetti, R., Liberatore, G., Offen, D., Korsmeyer, S.J., Przedborski, S. Bax ablation prevents dopaminergic neurodegeneration in the 1-methyl- 4-phenyl-1,2,3,6-tetrahydropyridine mouse model of Parkinson's disease. *Proc Natl Acad Sci U S A.* **2001,** *98*, 2837–42. doi: 10.1073/pnas.051633998
6. Johnson, M.D.; Xiang, H.; London, S.; Kinoshita, Y.; Knudson, M.; Mayberg, M.; Korsmeyer, S.J.; Morrison, R.S. Evidence for involvement of Bax and p53, but not caspases, in radiation-induced cell death of cultured postnatal hippocampal neurons. *J. Neurosci. Res.* **1998**, *54*, 721–733. https://doi.org/10.1002/(SICI)1097-4547(19981215)54:6<721::AID-JNR1>3.0.CO;2-1.
7. Miller, T.M.; Moulder, K.L.; Knudson, C.M.; Creedon, D.J.; Deshmukh, M.; Korsmeyer, S.J.; Johnson, E.M. Bax Deletion Further Orders the Cell Death Pathway in Cerebellar Granule Cells and Suggests a Caspase-independent Pathway to Cell Death. *J. Cell Biol.* **1997**, *139*, 205–217. https://doi.org/10.1083/jcb.139.1.205.
8. White, F.A.; Keller-Peck, C.R.; Knudson, C.M.; Korsmeyer, S.J.; Snider, W.D. Widespread Elimination of Naturally Occurring Neuronal Death inBax-Deficient Mice. *J. Neurosci.* **1998**, *18*, 1428–1439. https://doi.org/10.1523/jneurosci.18-04-01428.1998.
9. Kwon, Y.; Shin, J.; Nam, K.; An, J.S.; Yang, S.; Hong, S.; Bae, M.; Moon, K.; Cho, Y.; Woo, J.; et al. Rhizolutin, a Novel 7/10/6-Tricyclic Dilactone, Dissociates Misfolded Protein Aggregates and Reduces Apoptosis/Inflammation Associated with Alzheimer's Disease. *Angew. Chem. Int. Ed.* **2020**, *59*, 22994–22998. https://doi.org/10.1002/anie.202009294.
10. Rohn, T.T.; Kokoulina, P.; Eaton, C.R.; Poon, W.W. Caspase activation in transgenic mice with Alzheimer-like pathology: Results from a pilot study utilizing the caspase inhibitor, Q-VD-OPh. *Int. J. Clin. Exp. Med.* **2009**, *2*, 300–308.
11. Duan, W., Zhu, X., Ladenheim, B., Yu, Q.-S., Guo, Z., Oyler, J., Cutler, R.G., Cadet, J.L., Greig, N.H., Mattson, M.P. p53 inhibitors preserve dopamine neurons and motor function in experimental parkinsonism. *Ann Neurol*. **2002**, *52*, 597–606. https://doi.org/10.1002/ana.10350
12. Vila, M., Przedborski, S. Targeting programmed cell death in neurodegenerative diseases. *Nat Rev Neurosci.* **2003**, *4*, 365–375. https://doi.org/10.1038/nrn1100
13. Teismann, P.; Tieu, K.; Choi, D.-K.; Wu, D.-C.; Naini, A.; Hunot, S.; Vila, M.; Jackson-Lewis, V.; Przedborski, S. Cyclooxygenase-2 is instrumental in Parkinson's disease neurodegeneration. *Proc. Natl. Acad. Sci. USA* **2003**, *100*, 5473–5478. https://doi.org/10.1073/pnas.0837397100.
14. Du, Y., Ma, Z., Lin, S., Dodel, R. C., Gao, F., Bales, K. R., Triarhou, L. C., Chernet, E., Perry, K. W., Nelson, D. L., Luecke, S., Phebus, L. A., Bymaster, F. P., & Paul, S. M.. Minocycline prevents nigrostriatal dopaminergic neurodegeneration in the MPTP model of Parkinson's disease. *Proc Natl Acad Sci U S A.* **2001,** *98*, 14669–74
15. Kanthasamy, A.G.; Anantharam, V.; Zhang, D.; Latchoumycandane, C.; Jin, H.; Kaul, S.; Kanthasamy, A. A novel peptide inhibitor targeted to caspase-3 cleavage site of a proapoptotic kinase protein kinase C delta (PKCδ) protects against dopaminergic neuronal degeneration in Parkinson's disease models. *Free Radic. Biol. Med.* **2006**, *41*, 1578–1589. https://doi.org/10.1016/j.freeradbiomed.2006.08.016.
16. Ganjam, G.K.; Bolte, K.; Matschke, L.A.; Neitemeier, S.; Dolga, A.; Höllerhage, M.; Höglinger, G.; Adamczyk, A.; Decher, N.; Oertel, W.H.; et al. Mitochondrial damage by α-synuclein causes cell death in human dopaminergic neurons. *Cell Death Dis.* **2019**, *10*, 865. https://doi.org/10.1038/s41419-019-2091-2.
17. Ona, V., Li, M., Vonsattel, J. et al. Inhibition of caspase-1 slows disease progression in a mouse model of Huntington's disease. *Nature* **1999,** *399*, 263–267. https://doi.org/10.1038/20446
18. Aharony, I.; Ehrnhoefer, D.E.; Shruster, A.; Qiu, X.; Franciosi, S.; Hayden, M.; Offen, D. A Huntingtin-based peptide inhibitor of caspase-6 provides protection from mutant Huntingtin-induced motor and behavioral deficits. *Hum. Mol. Genet.* **2015**, *24*, 2604–2614. https://doi.org/10.1093/hmg/ddv023.
19. Li, M., Ona, V. O., Guégan, C., Chen, M., Jackson-Lewis, V., Andrews, L. J., Olszewski, A. J., Stieg, P. E., Lee, J. P., Przedborski, S., & Friedlander, R. M. Functional role of caspase-1 and caspase-3 in an ALS transgenic mouse model. *Science*. **2000**, *288*, 335–9. doi: 10.1126/science.288.5464.335.
20. Zhu, S., Stavrovskaya, I., Drozda, M. et al. Minocycline inhibits cytochrome c release and delays progression of amyotrophic lateral sclerosis in mice. *Nature* **2002,** *417*, 74–78 . https://doi.org/10.1038/417074a

21. Ahn, S.-W.; Jeon, G.S.; Kim, M.-J.; Shon, J.-H.; Kim, J.-E.; Shin, J.-Y.; Kim, S.-M.; Kim, S.H.; Ye, I.-H.; Lee, K.-W.; et al. Neuroprotective effects of JGK-263 in transgenic SOD1-G93A mice of amyotrophic lateral sclerosis. *J. Neurol. Sci.* **2014**, *340*, 112–116. https://doi.org/10.1016/j.jns.2014.03.008.
22. Hawking, Z.L. Alzheimer's disease: The role of mitochondrial dysfunction and potential new therapies: The International Journal of Student Research. *Biosci. Horizons* **2016**, *9*. hzw014. https://doi.org/10.1093/biohorizons/hzw014.
23. Wu, Y., Chen, M., & Jiang, J. Mitochondrial dysfunction in neurodegenerative diseases and drug targets via apoptotic signaling. *Mitochondrion*. **2019,** *49*, 35-45. doi: 10.1016/j.mito.2019.07.003.
24. Tewari, D., Stankiewicz, A. M., Mocan, A., Sah, A. N., Tzvetkov, N. T., Huminiecki, L., Horbańczuk, J. O., & Atanasov, A. G. Ethnopharmacological Approaches for Dementia Therapy and Significance of Natural Products and Herbal Drugs. *Front Aging Neurosci.* **2018**, *10*, 3. doi: 10.3389/fnagi.2018.00003.
25. Rajasekaran, A., Venkatasubramanian, G., Berk, M., & Debnath, M. Mitochondrial dysfunction in schizophrenia: pathways, mechanisms and implications. *Neurosci Biobehav Rev.* **2015**, *48*, 10-21. doi: 10.1016/j.neubiorev.2014.11.005.
26. Moreira, P.I.; Carvalho, C.; Zhu, X.; Smith, M.A.; Perry, G. Mitochondrial dysfunction is a trigger of Alzheimer's disease pathophysiology. *Biochim. Biophys. Acta Mol. Basis Dis.* **2010**, *1802*, 2–10. https://doi.org/10.1016/j.bbadis.2009.10.006.
27. Picone, P., Nuzzo, D., Caruana, L., Scafidi, V., & Di Carlo, M. Mitochondrial dysfunction: different routes to Alzheimer's disease therapy. *Oxid Med Cell Longev*. **2014**, 780179. doi: 10.1155/2014/780179.
28. Song, G., Li, Y., Lin, L., & Cao, Y. Anti-autophagic and anti-apoptotic effects of memantine in a SH-SY5Y cell model of Alzheimer's disease via mammalian target of rapamycin-dependent and -independent pathways. *Mol Med Rep* **2015,** *12*, 7615–7622
29. Sun, X.-Q.; Zhang, R.; Zhang, H.-D.; Yuan, P.; Wang, X.-J.; Zhao, Q.-H.; Wang, L.; Jiang, R.; Bogaard, H.J.; Jing, Z.-C. Reversal of right ventricular remodeling by dichloroacetate is related to inhibition of mitochondria-dependent apoptosis. *Hypertens. Res.* **2016**, *39*, 302–311. https://doi.org/10.1038/hr.2015.153.
30. Macdonald, R.; Barnes, K.; Hastings, C.; Mortiboys, H. Mitochondrial abnormalities in Parkinson's disease and Alzheimer's disease: Can mitochondria be targeted therapeutically? *Biochem. Soc. Trans.* **2018**, *46*, 891–909. https://doi.org/10.1042/bst20170501.
31. Park, J. S., Davis, R. L., & Sue, C. M. Mitochondrial Dysfunction in Parkinson's Disease: New Mechanistic Insights and Therapeutic Perspectives. Curr Neurol *Neurosci Rep*. **2018,** *18*, 21. doi: 10.1007/s11910-018-0829-3.
32. Flatters, S.J. The Contribution of Mitochondria to Sensory Processing and Pain. *Prog. Mol. Biol. Transl. Sci.* **2015**, *131*, 119–146. https://doi.org/10.1016/bs.pmbts.2014.12.004.
33. Smith, E.F.; Shaw, P.J.; De Vos, K.J. The role of mitochondria in amyotrophic lateral sclerosis. *Neurosci. Lett.* **2019**, *710*, 132933. https://doi.org/10.1016/j.neulet.2017.06.052.
34. Patergnani, S.; Fossati, V.; Bonora, M.; Giorgi, C.; Marchi, S.; Missiroli, S.; Rusielewicz, T.; Wieckowski, M.; Pinton, P. Mitochondria in Multiple Sclerosis: Molecular Mechanisms of Pathogenesis. *Int. Rev. Cell. Mol. Biol.* **2017**, *328*, 49–103. https://doi.org/10.1016/bs.ircmb.2016.08.003.
35. Marchionini, D.M.; Collier, T.J.; Pitzer, M.R.; Sortwell, C.E. Reassessment of caspase inhibition to augment grafted dopamine neuron survival. *Cell Transplant.* **2004**, *13*, 273–282. https://doi.org/10.3727/000000004783983972.
36. Gray, J., Haran, M. M., Schneider, K., Vesce, S., Ray, A. M., Owen, D., White, I. R., Cutler, P., & Davis, J. B. Evidence that inhibition of cathepsin-B contributes to the neuroprotective properties of caspase inhibitor Tyr-Val-Ala-Asp-chloromethyl ketone. *J Biol Chem*. **2001,** *276*, 32750–5. doi: 10.1074/jbc.M103150200.
37. Sivasangari, K.; Rajan, K.E. Standardized *Bacopa monnieri* Extract Ameliorates Learning and Memory Impairments through Synaptic Protein, Neurogranin, Pro-and Mature BDNF Signaling, and HPA Axis in Prenatally Stressed Rat Offspring. *Antioxidants* **2020**, *9*, 1229. https://doi.org/10.3390/antiox9121229.
38. Lee, J.-E.; Sim, H.; Yoo, H.M.; Lee, M.; Baek, A.; Jeon, Y.-J.; Seo, K.-S.; Son, M.-Y.; Yoon, J.S.; Kim, J. Neuroprotective Effects of Cryptotanshinone in a Direct Reprogramming Model of Parkinson's Disease. *Molecules* **2020**, *25*, 3602. https://doi.org/10.3390/molecules25163602.
39. Pillai, A., Veeranan-Karmegam, R., Dhandapani, K. M., & Mahadik, S. P. Cystamine prevents haloperidol-induced decrease of BDNF/TrkB signaling in mouse frontal cortex. *J Neurochem*. **2008,** *107*, 941–51. doi: 10.1111/j.1471-4159.2008.05665.x.
40. Eckert, G. P., Chang, S., Eckmann, J., Copanaki, E., Hagl, S., Hener, U., Müller, W. E., & Kögel, D. Liposome-incorporated DHA increases neuronal survival by enhancing non-amyloidogenic APP processing. *Biochim Biophys Acta*. **2011**, *1808*, 236–43. doi: 10.1016/j.bbamem.2010.10.014.
41. Ravishankar, D.; Corona, G.; Hogan, S.M.; Spencer, J.P.; Greco, F.; Osborn, H.M. Thioflavones as novel neuroprotective agents. *Bioorganic Med. Chem.* **2016**, *24*, 5513–5520. https://doi.org/10.1016/j.bmc.2016.09.006.
42. Pillai, A., Dhandapani, K. M., Pillai, B. A., Terry, A. V., Jr, & Mahadik, S. P. Erythropoietin prevents haloperidol treatment-induced neuronal apoptosis through regulation of BDNF. *Neuropsychopharmacology*. **2008**, *33*, 1942-51. doi: 10.1038/sj.npp.1301566.
43. Li, H., Wang, B., Zhu, C., Feng, Y., Wang, S., Shahzad, M., Hu, C., Mo, M., Du, F., & Yu, X. 17β-estradiol impedes Bax-involved mitochondrial apoptosis of retinal nerve cells induced by oxidative damage via the phosphatidylinositol 3-kinase/Akt signal pathway. *J Mol Neurosci.* **2013,** *50*, 482-93. doi: 10.1007/s12031-013-9968-9.

44. Nuñez-Figueredo, Y.; Pardo-Andreu, G.L.; Ramírez-Sánchez, J.; Delgado-Hernández, R.; Ochoa-Rodríguez, E.; Verdecia-Reyes, Y.; Naal, Z.; Muller, A.P.; Portela, L.V.; Souza, D.O. Antioxidant effects of JM-20 on rat brain mitochondria and synaptosomes: Mitoprotection against Ca2+-induced mitochondrial impairment. *Brain Res. Bull.* **2014**, *109*, 68–76. https://doi.org/10.1016/j.brainresbull.2014.10.001.
45. Momeni, H.R.; Kanje, M. Calpain inhibitors delay injury-induced apoptosis in adult mouse spinal cord motor neurons. *Neuroreport* **2006**, *17*, 761–765. https://doi.org/10.1097/01.wnr.0000220127.01597.04.
46. Liu, W., Jalewa, J., Sharma, M., Li, G., Li, L., & Hölscher, C. Neuroprotective effects of lixisenatide and liraglutide in the 1-methyl-4-phenyl-1,2,3,6-tetrahydropyridine mouse model of Parkinson's disease. *Neuroscience*. **2015,** *303*, 42-50. doi: 10.1016/j.neuroscience.2015.06.054.
47. Manigandan, V.; Nataraj, J.; Karthik, R.; Manivasagam, T.; Saravanan, R.; Thenmozhi, A.J.; Essa, M.M.; Guillemin, G.J. Low Molecular Weight Sulfated Chitosan: Neuroprotective Effect on Rotenone-Induced In Vitro Parkinson's Disease. *Neurotox. Res.* **2019**, *35*, 505–515. https://doi.org/10.1007/s12640-018-9978-z.
48. Sharif, R., Aghsami, M., Gharghabi, M., Sanati, M., Khorshidahmad, T., Vakilzadeh, G., Mehdizadeh, H., Gholizadeh, S., Taghizadeh, G., & Sharifzadeh, M. Melatonin reverses H-89 induced spatial memory deficit: Involvement of oxidative stress and mitochondrial function. *Behav Brain Res*. **2017**, *316*, 115-124. doi: 10.1016/j.bbr.2016.08.040.
49. Casarejos, M.J.; Menéndez, J.; Solano, R.M.; Rodríguez-Navarro, J.A.; De Yébenes, J.G.; Mena, M.A. Susceptibility to rotenone is increased in neurons from parkin null mice and is reduced by minocycline. *J. Neurochem.* **2006**, *97*, 934–946. https://doi.org/10.1111/j.1471-4159.2006.03777.x.
50. Sirianni, A.C.; Jiang, J.; Zeng, J.; Mao, L.L.; Zhou, S.; Sugarbaker, P.; Zhang, X.; Li, W.; Friedlander, R.M.; Wang, X. N-acetyl-l -tryptophan, but not N-acetyl-d -tryptophan, rescues neuronal cell death in models of amyotrophic lateral sclerosis. *J. Neurochem.* **2015**, *134*, 956–968. https://doi.org/10.1111/jnc.13190.
51. Ullah, N., Ullah, I., Lee, H. Y., Naseer, M. I., Seok, P. M., Ahmed, J., & Kim, M. O. Protective function of nicotinamide against ketamine-induced apoptotic neurodegeneration in the infant rat brain. *J Mol Neurosci*. **2012,** *47*, 67-75. doi: 10.1007/s12031-011-9685-1.
52. Mookherjee, P.; Johnson, G.V. Tau phosphorylation during apoptosis of human SH-SY5Y neuroblastoma cells. *Brain Res.* **2001**, *921*, 31–43. https://doi.org/10.1016/s0006-8993(01)03074-8.
53. Liu, W., Li, Y., Jalewa, J., Saunders-Wood, T., Li, L., & Hölscher, C. Neuroprotective effects of an oxyntomodulin analogue in the MPTP mouse model of Parkinson's disease. *Eur J Pharmacol.* **2015**, *765*, 284-90. doi: 10.1016/j.ejphar.2015.08.038.
54. Gu, C.; Zhang, Y.; Hu, Q.; Wu, J.; Ren, H.; Liu, C.-F.; Wang, G. P7C3 inhibits GSK3β activation to protect dopaminergic neurons against neurotoxin-induced cell death in vitro and in vivo. *Cell Death Dis.* **2017**, *8*, e2858. https://doi.org/10.1038/cddis.2017.250.
55. Hu, X.; Weng, Z.; Chu, C.; Zhang, L.; Cao, G.; Gao, Y.; Signore, A.; Zhu, J.; Hastings, T.; Greenamyre, J.T.; et al. Peroxiredoxin-2 Protects against 6-Hydroxydopamine-Induced Dopaminergic Neurodegeneration via Attenuation of the Apoptosis Signal-Regulating Kinase (ASK1) Signaling Cascade. *J. Neurosci.* **2011**, *31*, 247–261. https://doi.org/10.1523/jneurosci.4589-10.2011.
56. Squitieri, F., Di Pardo, A., Favellato, M., Amico, E., Maglione, V., Frati, L. Pridopidine, a dopamine stabilizer, improves motor performance and shows neuroprotective effects in Huntington disease R6/2 mouse model. *J Cell Mol Med*, **2015,** *19*, 2540–2548. doi: 10.1111/jcmm.12604.
57. Selvakumar, K.; Bavithra, S.; Suganthi, M.; Benson, C.S.; Elumalai, P.; Arunkumar, R.; Krishnamoorthy, G.; Venkataraman, P.; Arunakaran, J. Protective Role of Quercetin on PCBs-Induced Oxidative Stress and Apoptosis in Hippocampus of Adult Rats. *Neurochem. Res.* **2012**, *37*, 708–721. https://doi.org/10.1007/s11064-011-0661-5.
58. Cheng, B., Martinez, A.A., Morado, J., Scofield, V., Roberts, J.L., Maffi, S.K. Retinoic acid protects against proteasome inhibition associated cell death in SH-SY5Y cells via the AKT pathway. *Neurochem Int*, **2012,** *62*, 31–42. doi: 10.1016/j.neuint.2012.10.014.
59. Duarte-Silva, E., Araújo, S., Oliveira, W. H., Lós, D. B., França, M., Bonfanti, A. P., Peron, G., Thomaz, L. L., Verinaud, L., Nunes, A., & Peixoto, C. A. Sildenafil ameliorates EAE by decreasing apoptosis in the spinal cord of C57BL/6 mice. *J Neuroimmunol.* **2018,** *321*, 125-137. doi: 10.1016/j.jneuroim.2018.06.002.
60. Tai, J., Liu, W., Li, Y., Li, L., Hölscher, C. Neuroprotective effects of a triple GLP-1/GIP/glucagon receptor agonist in the APP/PS1 transgenic mouse model of Alzheimer's disease. *Brain Res*, **2017,** *1678*, 64–74. doi: 10.1016/j.brainres.2017.10.012.
61. Timpe, J.M., Wang, C.Z., Kim, J., Johnson, K.M. α-amino-3-hydroxy-5-methyl-4-isoxazoleproprionic acid receptor activation protects against phencyclidine-induced caspase-3 activity by activating voltage-gated calcium channels. *J Neurosci Res*. **2014**, *92*, 1785–1791. doi: 10.1002/jnr.23446.
62. Shoshan-Barmatz, V.; Nahon-Crystal, E.; Shteinfer-Kuzmine, A.; Gupta, R. VDAC1, mitochondrial dysfunction, and Alzheimer's disease. *Pharmacol. Res.* **2018**, *131*, 87–101. https://doi.org/10.1016/j.phrs.2018.03.010.
63. Deleglise, B.; Lassus, B.; Soubeyre, V.; Alleaume-Butaux, A.; Hjorth, J.J.; Vignes, M.; Schneider, B.; Brugg, B.; Viovy, J.-L.; Peyrin, J.-M. Synapto-Protective Drugs Evaluation in Reconstructed Neuronal Network. *PLoS ONE* **2013**, *8*, e71103. https://doi.org/10.1371/journal.pone.0071103.

64. Liu, Y.; Wang, W.; Li, Y.; Xiao, Y.; Cheng, J.; Jia, J. The 5-Lipoxygenase Inhibitor Zileuton Confers Neuroprotection against Glutamate Oxidative Damage by Inhibiting Ferroptosis. *Biol. Pharm. Bull.* **2015**, *38*, 1234–1239. https://doi.org/10.1248/bpb.b15-00048.
65. Gómez, C.; Reiriz, J.; Piqué, M.; Gil, J.; Ferrer, I.; Ambrosio, S. Low concentrations of 1-methyl-4-phenylpyridinium ion induce caspase-mediated apoptosis in human SH-SY5Y neuroblastoma cells. *J. Neurosci. Res.* **2001**, *63*, 421–428. https://doi.org/10.1002/1097-4547(20010301)63:5<421::AID-JNR1037>3.0.CO;2-4.
66. Thompson, C.K., Brenowitz, E.A. Caspase inhibitor infusion protects an avian song control circuit from seasonal-like neurodegeneration. *J Neurosci*, **2008,** *28,* 7130–7136. doi: 10.1523/JNEUROSCI.0663-08.2008.
67. Gallego-Sandín, S., Novalbos, J., Rosado, A., Cano-Abad, M. F., Arias, E., Abad-Santos, F., & García, A. G. Albumin prevents mitochondrial depolarization and apoptosis elicited by endoplasmic reticulum calcium depletion of neuroblastoma cells. *Eur J Pharmacol*. **2005,** *520*, 1-11. doi: 10.1016/j.ejphar.2005.06.044.
68. Mukhopadhyay, D.; Hammami, M.; Khalouf, A.; Al Shaikh, Y.; Mohammed, A.K.; Hamad, M.; Salehi, A.; Taneera, J. Dimethyloxalylglycine (DMOG) and the Caspase Inhibitor "Ac-LETD-CHO" Protect Neuronal ND7/23 Cells of Gluocotoxicity. *Exp. Clin. Endocrinol. Diabetes* **2021**, *129*, 420–428. https://doi.org/10.1055/a-0919-4489.
69. Ullah, I.; Park, H.Y.; Kim, M.O. Anthocyanins Protect against Kainic Acid-induced Excitotoxicity and Apoptosis via ROS-activated AMPK Pathway in Hippocampal Neurons. *CNS Neurosci. Ther.* **2014**, *20*, 327–338. https://doi.org/10.1111/cns.12218.
70. Nataraj, J., Manivasagam, T., Justin Thenmozhi, A., Essa, M.M. Neuroprotective effect of asiatic acid on rotenone-induced mitochondrial dysfunction and oxidative stress-mediated apoptosis in differentiated SH-SYS5Y cells. *Nutr Neurosci*, **2017,** *20*, 351–359. doi: 10.1080/1028415X.2015.1135559.
71. Song, D.; Jiang, X.; Liu, Y.; Sun, Y.; Cao, S.; Zhang, Z. Asiaticoside Attenuates Cell Growth Inhibition and Apoptosis Induced by Aβ1-42 via Inhibiting the TLR4/NF-κB Signaling Pathway in Human Brain Microvascular Endothelial Cells. *Front. Pharmacol.* **2018**, *9*, 28. https://doi.org/10.3389/fphar.2018.00028.
72. Werth, J.L.; Deshmukh, M.; Cocabo, J.; Johnson, E.M.; Rothman, S.M. Reversible Physiological Alterations in Sympathetic Neurons Deprived of NGF but Protected from Apoptosis by Caspase Inhibition or Bax Deletion. *Exp. Neurol.* **2000**, *161*, 203–211. https://doi.org/10.1006/exnr.1999.7241.
73. Jung, E.B.; Lee, C.S. Baicalein attenuates proteasome inhibition-induced apoptosis by suppressing the activation of the mitochondrial pathway and the caspase-8- and Bid-dependent pathways. *Eur. J. Pharmacol.* **2014**, *730*, 116–124. https://doi.org/10.1016/j.ejphar.2014.02.039.
74. Liu, X.-Y., Zhang, L.-J., Chen, Z., Liu, L.-B. The PTEN inhibitor bpV(pic) promotes neuroprotection against amyloid β-peptide (25-35)-induced oxidative stress and neurotoxicity. *Neurol Res*, **2017,** *39,* 758–765. doi: 10.1080/01616412.2017.1317916.
75. Gliyazova, N.S.; Ibeanu, G.C. The Chemical Molecule B355252 is Neuroprotective in an In Vitro Model of Parkinson's Disease. *Cell. Mol. Neurobiol.* **2016**, *36*, 1109–1122. https://doi.org/10.1007/s10571-015-0304-5.
76. Silva, J.; Alves, C.; Pinteus, S.; Mendes, S.; Pedrosa, R. Seaweeds' neuroprotective potential set in vitro on a human cellular stress model. *Mol. Cell. Biochem.* **2020**, *473*, 229–238. https://doi.org/10.1007/s11010-020-03824-5.
77. Sagot, Y., Toni, N., Perrelet, D., Lurot, S., King, B., Rixner, H., Mattenberger, L., Waldmeier, P. C., & Kato, A. C. An orally active anti-apoptotic molecule (CGP 3466B) preserves mitochondria and enhances survival in an animal model of motoneuron disease. *Br J Pharmacol*. **2000,** *131*, 721-8. doi: 10.1038/sj.bjp.0703633.
78. Tseng, Y.-T.; Tsai, Y.-H.; Fülöp, F.; Chang, F.-R.; Lo, Y.-C. 2-Iodo-4′-Methoxychalcone Attenuates Methylglyoxal-Induced Neurotoxicity by Activation of GLP-1 Receptor and Enhancement of Neurotrophic Signal, Antioxidant Defense and Glyoxalase Pathway. *Molecules* **2019**, *24*, 2249. https://doi.org/10.3390/molecules24122249.
79. Zhang, X.; Boulton, A.A.; Zuo, D.-M.; Yu, P.H. MK-801 induces apoptotic neuronal death in the rat retrosplenial cortex: Prevention by cycloheximide and R(?)-2-Hexyl-N- Methylpropargylamine. *J. Neurosci. Res.* **1996**, *46*, 82–89. https://doi.org/10.1002/(sici)1097-4547(19961001)46:1<82::aid-jnr10>3.0.co;2-e.
80. El-Sahar, A. E., Rastanawi, A. A., El-Yamany, M. F., Saad, M. A. Dapagliflozin improves behavioral dysfunction of Huntington's disease in rats via inhibiting apoptosis-related glycolysis. *Life Sci*, **2020**, *257*, 118076. doi: 10.1016/j.lfs.2020.118076.
81. Rosenstock, T. R., de Brito, O. M., Lombardi, V., Louros, S., Ribeiro, M., Almeida, S., Ferreira, I. L., Oliveira, C. R., & Rego, A. C. FK506 ameliorates cell death features in Huntington's disease striatal cell models. *Neurochem Int*. **2011**, *59*, 600-9. doi: 10.1016/j.neuint.2011.04.009.
82. Huang, C.; Lin, Y.; Su, H.; Ye, D. Forsythiaside Protects Against Hydrogen Peroxide-Induced Oxidative Stress and Apoptosis in PC12 Cell. *Neurochem. Res.* **2015**, *40*, 27–35. https://doi.org/10.1007/s11064-014-1461-5.
83. Wei, H., Gao, Z., Zheng, L., Zhang, C., Liu, Z., Yang, Y., Teng, H., Hou, L., Yin, Y., & Zou, X. Protective Effects of Fucoidan on Aβ25-35 and d-Gal-Induced Neurotoxicity in PC12 Cells and d-Gal-Induced Cognitive Dysfunction in Mice. *Mar Drugs*. **2017,** *15*, 77. doi: 10.3390/md15030077.
84. Song, D.H.; Kim, G.-J.; Lee, K.J.; Shin, J.S.; Kim, D.-H.; Park, B.-J.; An, J.H. Mitigation Effects of a Novel Herbal Medicine, Hepad, on Neuroinflammation, Neuroapoptosis, and Neuro-Oxidation. *Molecules* **2018**, *23*, 2920. https://doi.org/10.3390/molecules23112920.

85. Peixoto, P.M.; Teijido, O.; Mirzalieva, O.; Dejean, L.M.; Pavlov, E.V.; Antonsson, B.; Kinnally, K.W. MAC inhibitors antagonize the pro-apoptotic effects of tBid and disassemble Bax / Bak oligomers. *J. Bioenerg. Biomembr.* **2017**, *49*, 65–74. https://doi.org/10.1007/s10863-015-9635-7.
86. Zhang, J., Zhang, Z., Bao, J., Yu, Z., Cai, M., Li, X., Wu, T., Xiang, J., & Cai, D.
87. Wong-Guerra, M., Jiménez-Martin, J., Fonseca-Fonseca, L. A., Ramírez-Sánchez, J., Montano-Peguero, Y., Rocha, J. B., D Avila, F., de Assis, A. M., Souza, D. O., Pardo-Andreu, et al. JM-20 protects memory acquisition and consolidation on scopolamine model of cognitive impairment. *Neurol Res.* **2019**, *41*, 385-398. doi: 10.1080/01616412.2019.1573285.
88. Busquets, O.; Ettcheto, M.; Verdaguer, E.; Castro-Torres, R.D.; Auladell, C.; Beas-Zarate, C.; Folch, J.; Camins, A. JNK1 inhibition by Licochalcone A leads to neuronal protection against excitotoxic insults derived of kainic acid. *Neuropharmacology* **2018**, *131*, 440–452. https://doi.org/10.1016/j.neuropharm.2017.10.030.
89. Xian, H.; Zhao, J.; Zheng, Y.; Wang, M.; Huang, J.; Wu, B.; Sun, C.; Yang, Y. MADP, a salidroside analog, protects hippocampal neurons from glutamate induced apoptosis. *Life Sci.* **2014**, *103*, 34–40. https://doi.org/10.1016/j.lfs.2014.02.040.
90. Zhou, L., Flores, J., Noël, A., Beauchet, O., Sjöström, P. J., & LeBlanc, A. C. Methylene blue inhibits Caspase-6 activity, and reverses Caspase-6-induced cognitive impairment and neuroinflammation in aged mice. *Acta Neuropathol Commun.* **2019**, *7,* 210. doi: 10.1186/s40478-019-0856-6.
91. El-Mir, M.-Y.; Detaille, D.; R-Villanueva, G.; Esteban, M.D.; Guigas, B.; Attia, S.; Fontaine, E.; Almeida, A.; Leverve, X. Neuroprotective Role of Antidiabetic Drug Metformin Against Apoptotic Cell Death in Primary Cortical Neurons. *J. Mol. Neurosci.* **2008**, *34*, 77–87. https://doi.org/10.1007/s12031-007-9002-1.
92. Zhang, X., Rojas, J. C., & Gonzalez-Lima, F. Methylene blue prevents neurodegeneration caused by rotenone in the retina. *Neurotox Res*s. **2006,** *9,* 47-57. doi: 10.1007/BF03033307
93. Rojas, J.C.; John, J.M.; Lee, J.; Gonzalez-Lima, F. Methylene Blue Provides Behavioral and Metabolic Neuroprotection Against Optic Neuropathy. *Neurotox. Res.* **2009**, *15*, 260–273. https://doi.org/10.1007/s12640-009-9027-z.
94. Wang, C.; McInnis, J.; West, J.B.; Bao, J.; Anastasio, N.; Guidry, J.A.; Ye, Y.; Salvemini, D.; Johnson, K.M. Blockade of Phencyclidine-Induced Cortical Apoptosis and Deficits in Prepulse Inhibition by M40403, a Superoxide Dismutase Mimetic. *J. Pharmacol. Exp. Ther.* **2003**, *304*, 266–271. https://doi.org/10.1124/jpet.102.041798.
95. Bonelli, R. M., Hödl, A. K., Hofmann, P., & Kapfhammer, H. P. Neuroprotection in Huntington's disease: a 2-year study on minocycline. *Int Clin Psychopharmacol*. **2004**, *19*, 337-42. doi: 10.1097/00004850-200411000-00004.
96. Miyaoka, T., Yasukawa, R., Yasuda, H., Hayashida, M., Inagaki, T., & Horiguchi, J. Minocycline as adjunctive therapy for schizophrenia: an open-label study. *Clin Neuropharmacol*. **2008**, *31*, 287-92. doi: 10.1097/WNF.0b013e3181593d45.
97. Månsson, R., Hansson, M. J., Morota, S., Uchino, H., Ekdahl, C. T., & Elmér, E. Re-evaluation of mitochondrial permeability transition as a primary neuroprotective target of minocycline. *Neurobiol Dis*. **2007**, *25*, 198-205. doi: 10.1016/j.nbd.2006.09.008.
98. Kumar, S.N.K.; Deepthy, J.; Saraswathi, U.; Thangarajeswari, M.; Kanna, S.Y.; Ezhil, P.; Kalaiselvi, P. *Morinda citrifolia* mitigates rotenone-induced striatal neuronal loss in male Sprague-Dawley rats by preventing mitochondrial pathway of intrinsic apoptosis. *Redox Rep.* **2017**, *22*, 418–429. https://doi.org/10.1080/13510002.2016.1253449.
99. Gopinath, K., Prakash, D., & Sudhandiran, G. Neuroprotective effect of naringin, a dietary flavonoid against 3-nitropropionic acid-induced neuronal apoptosis. *Neurochem Int.* **2011**, *59,* 1066-73. doi: 10.1016/j.neuint.2011.08.022.
100. Liu, W.-B.; Zhou, J.; Qu, Y.; Li, X.; Lu, C.-T.; Xie, K.-L.; Sun, X.-L.; Fei, Z. Neuroprotective effect of osthole on MPP+-induced cytotoxicity in PC12 cells via inhibition of mitochondrial dysfunction and ROS production. *Neurochem. Int.* **2010**, *57*, 206–215. https://doi.org/10.1016/j.neuint.2010.05.011.
101. De Sarno, P.; Shestopal, S.A.; King, T.D.; Zmijewska, A.; Song, L.; Jope, R.S. Muscarinic Receptor Activation Protects Cells from Apoptotic Effects of DNA Damage, Oxidative Stress, and Mitochondrial Inhibition. *J. Biol. Chem.* **2003**, *278*, 11086–11093. https://doi.org/10.1074/jbc.m212157200.
102. Dong, H.; Li, R.; Yu, C.; Xu, T.; Zhang, X.; Dong, M. Paeoniflorin inhibition of 6-hydroxydopamine-induced apoptosis in PC12 cells via suppressing reactive oxygen species-mediated PKCδ/NF-κB pathway. *Neuroscience* **2015**, *285*, 70–80. https://doi.org/10.1016/j.neuroscience.2014.11.008.
103. Wang, D., Wong, H. K., Feng, Y. B., & Zhang, Z. J. Paeoniflorin, a natural neuroprotective agent, modulates multiple anti-apoptotic and pro-apoptotic pathways in differentiated PC12 cells. *Cell Mol Neurobiol*. **2013,** *33,* 521–529. doi: 10.1007/s10571-013-9914-y.
104. Sanz, E.; Quintana, A.; Battaglia, V.; Toninello, A.; Hidalgo, J.; Ambrosio, S.; Valoti, M.; Marco, J.L.; Tipton, K.F.; Unzeta, M. Anti-apoptotic effect of Mao-B inhibitor PF9601N [N-(2-propynyl)-2-(5-benzyloxy-indolyl) methylamine] is mediated by p53 pathway inhibition in MPP+-treated SH-SY5Y human dopaminergic cells. *J. Neurochem.* **2008**, *105*, 2404–2417. https://doi.org/10.1111/j.1471-4159.2008.05326.x.
105. Hong, S.-Y.; Jeong, W.-S.; Jun, M. Protective Effects of the Key Compounds Isolated from Corni fructus against β-Amyloid-Induced Neurotoxicity in PC12 Cells. *Molecules* **2012**, *17*, 10831–10845. https://doi.org/10.3390/molecules170910831.
106. Yang, L.; Sugama, S.; Mischak, R.P.; Kiaei, M.; Bizat, N.; Brouillet, E.; Joh, T.H.; Beal, M.F. A novel systemically active caspase inhibitor attenuates the toxicities of MPTP, malonate, and 3NP in vivo. *Neurobiol. Dis.* **2004**, *17*, 250–259. https://doi.org/10.1016/j.nbd.2004.07.021.

107. Dionne, K.R.; Leser, J.S.; Lorenzen, K.A.; Beckham, J.D.; Tyler, K.L. A brain slice culture model of viral encephalitis reveals an innate CNS cytokine response profile and the therapeutic potential of caspase inhibition. *Exp. Neurol.* **2011**, *228*, 222–231. https://doi.org/10.1016/j.expneurol.2011.01.006.
108. Wu, J., Wang, Y., Liang, S., Ma, H. Cytoprotective effect of selective small-molecule caspase inhibitors against staurosporine-induced apoptosis. *Drug Des Devel Ther*, **2014,** *8*, 583–600. doi: 10.2147/DDDT.S60283.
109. Sifringer, M.; Bendix, I.; Borner, C.; Endesfelder, S.; Von Haefen, C.; Kalb, A.; Holifanjaniaina, S.; Prager, S.; Schlager, G.W.; Keller, M.; et al. Prevention of neonatal oxygen-induced brain damage by reduction of intrinsic apoptosis. *Cell Death Dis.* **2012**, *3*, e250. https://doi.org/10.1038/cddis.2011.133.
110. Lee, C.S.; Han, E.S.; Han, Y.S.; Bang, H. Differential effect of calmodulin antagonists on MG132-induced mitochondrial dysfunction and cell death in PC12 cells. *Brain Res. Bull.* **2005**, *67*, 225–234. https://doi.org/10.1016/j.brainresbull.2005.07.003.
111. Rahimian, R., Fakhfouri, G., Ejtemaei Mehr, S., Ghia, J. E., Genazzani, A. A., Payandemehr, B., Dehpour, A. R., Mousavizadeh, K., & Lim, D. Tropisetron attenuates amyloid-beta-induced inflammatory and apoptotic responses in rats. *Eur J Clin Invest.* **2013,** *43*, 1039–51. doi: 10.1111/eci.12141.
112. Kim, I.S.; Choi, D.-K.; Jung, H.J. Neuroprotective Effects of Vanillyl Alcohol in Gastrodia elata Blume Through Suppression of Oxidative Stress and Anti-Apoptotic Activity in Toxin-Induced Dopaminergic MN9D Cells. *Molecules* **2011**, *16*, 5349–5361. https://doi.org/10.3390/molecules16075349.
113. Inoue, H., Tsukita, K., Iwasato, T., Suzuki, Y., Tomioka, M., Tateno, M., Nagao, M., Kawata, A., Saido, T. C., Miura, M., Misawa, H., Itohara, S., & Takahashi, R. The crucial role of caspase-9 in the disease progression of a transgenic ALS mouse model. *EMBO J.* **2003**, *22*, 6665–74. doi: 10.1093/emboj/cdg634.
114. Bizat, N., Galas, M. C., Jacquard, C., Boyer, F., Hermel, J. M., Schiffmann, S. N., Hantraye, P., Blum, D., & Brouillet, E. Neuroprotective effect of zVAD against the neurotoxin 3-nitropropionic acid involves inhibition of calpain. *Neuropharmacology*. **2005**, *49*, 695–702. doi: 10.1016/j.neuropharm.2005.04.030.
115. Perche, O.; Doly, M.; Ranchon-Cole, I. Transient protective effect of caspase inhibitors in RCS rat. *Exp. Eye Res.* **2008**, *86*, 519–527. https://doi.org/10.1016/j.exer.2007.12.005.
116. Cutillas, B.; Espejo, M.; Gil, J.; Ferrer, I.; Ambrosio, S. Caspase inhibition protects nigral neurons against 6-OHDA-induced retrograde degeneration. *Neuroreport* **1999**, *10*, 2605–2608. https://doi.org/10.1097/00001756-199908200-00030.
117. Chen, W.-F.; Chakraborty, C.; Sung, C.-S.; Feng, C.-W.; Jean, Y.-H.; Lin, Y.-Y.; Hung, H.-C.; Huang, T.-Y.; Huang, S.-Y.; Su, T.-M.; et al. Neuroprotection by marine-derived compound, 11-dehydrosinulariolide, in an in vitro Parkinson's model: A promising candidate for the treatment of Parkinson's disease. *Naunyn-Schmiedeberg's Arch. Pharmacol.* **2012**, *385*, 265–275. https://doi.org/10.1007/s00210-011-0710-2.
118. Bozzatello, P.; Brignolo, E.; De Grandi, E.; Bellino, S. Supplementation with Omega-3 Fatty Acids in Psychiatric Disorders: A Review of Literature Data. *J. Clin. Med.* **2016**, *5*, 67. https://doi.org/10.3390/jcm5080067.
119. Erythropoietin for Cognitive Deficits Associated with Schizophrenia, Bipolar Disorder, and Major Depression: A Systematic Review. *Pharmacopsychiatry*. **2018,** *51*, 100–104. doi: 10.1055/s-0043-114670.
120. Weiser, M., Levi, L., Zamora, D., Biegon, A., SanGiovanni, J. P., Davidson, M., Burshtein, S., Gonen, I., Radu, P., Slobozean Pavalache, K., Nastas, I., Hemi, R., Ryan, T., & Davis, J. M. Effect of Adjunctive Estradiol on Schizophrenia Among Women of Childbearing Age: A Randomized Clinical Trial. *JAMA Psychiatry.* **2019**, *76*, 1009–1017. doi: 10.1001/jamapsychiatry.2019.1842.
121. Schwartz, D.L. Quercetin as an Augmentation Agent in Schizophrenia. *J Clin Psychopharmacol*. **2016,** *36*, 282–3. doi: 10.1097/JCP.0000000000000498.
122. Ben-Azu, B.; Aderibigbe, A.O.; Omogbiya, I.A.; Ajayi, A.M.; Iwalewa, E.O. Morin Pretreatment Attenuates Schizophrenia-Like Behaviors in Experimental Animal Models. *Drug Res.* **2018**, *68*, 159–167. https://doi.org/10.1055/s-0043-119127.
123. Morera-Fumero, A.L.; Abreu-Gonzalez, P. Diazepam Discontinuation Through Agomelatine in Schizophrenia With Insomnia and Depression. *J. Clin. Psychopharmacol.* **2010**, *30*, 739–741. https://doi.org/10.1097/jcp.0b013e3181fbbb7d.
124. Shamir, E.; Laudon, M.; Barak, Y.; Anis, Y.; Rotenberg, V.; Elizur, A.; Zisapel, N. Melatonin Improves Sleep Quality of Patients With Chronic Schizophrenia. *J. Clin. Psychiatry* **2000**, *61*, 373–377. https://doi.org/10.4088/jcp.v61n0509.
125. Shamir, E.; Barak, Y.; Shalman, I.; Laudon, M.; Zisapel, N.; Tarrasch, R.; Elizur, A.; Weizman, R. Melatonin Treatment for Tardive Dyskinesia: A double-blind, placebo-controlled, crossover study. *Arch. Gen. Psychiatry* **2001**, *58*, 1049–1052. https://doi.org/10.1001/archpsyc.58.11.1049.
126. Hassanpour, F.; Zarghami, M.; Mouodi, S.; Moosazadeh, M.; Barzegar, F.; Bagheri, M.; Hendouei, N. Adjunctive Memantine Treatment of Schizophrenia: A Double-Blind, Randomized Placebo-Controlled Study. *J. Clin. Psychopharmacol.* **2019**, *39*, 634–638. https://doi.org/10.1097/jcp.0000000000001115.
127. Zhang, L., Zheng, H., Wu, R., Kosten, T. R., Zhang, X. Y., & Zhao, J. The effect of minocycline on amelioration of cognitive deficits and pro-inflammatory cytokines levels in patients with schizophrenia. *Schizophr Res*. **2019** *212*, 92–98. doi: 10.1016/j.schres.2019.08.005.
128. Sepehrmanesh, Z.; Heidary, M.; Akasheh, N.; Akbari, H.; Heidary, M. Therapeutic effect of adjunctive N-acetyl cysteine (NAC) on symptoms of chronic schizophrenia: A double-blind, randomized clinical trial. *Prog. Neuro-Psychopharmacol. Biol. Psychiatry* **2018**, *82*, 289–296. https://doi.org/10.1016/j.pnpbp.2017.11.001.

129. Park, J.H.; Kang, S.-S.; Kim, J.Y.; Tchah, H. The Antioxidant N-Acetylcysteine Inhibits Inflammatory and Apoptotic Processes in Human Conjunctival Epithelial Cells in a High-Glucose Environment. *Investig. Opthalmology Vis. Sci.* **2015**, *56*, 5614–5621. https://doi.org/10.1167/iovs.15-16909.
130. A Wittkopp, T.; Abuzzahab, F.S. Nicotinic acid and nicotinamide adenine dinucleotide (NAD) therapy in schizophrenia: A review. *Behav. Neuropsychiatry* **1972**, *4*, 6–12.
131. Lerner, V.; Miodownik, C.; Gibel, A.; Sirota, P.; Bush, I.; Elliot, H.; Benatov, R.; Ritsner, M.S. The Retinoid X Receptor Agonist Bexarotene Relieves Positive Symptoms of Schizophrenia. *J. Clin. Psychiatry* **2013**, *74*, 1224–1232. https://doi.org/10.4088/jcp.12m08160.
132. Goff, D. C., Cather, C., Freudenreich, O., Henderson, D. C., Evins, A. E., Culhane, M. A., & Walsh, J. P. A placebo-controlled study of sildenafil effects on cognition in schizophrenia. *Psychopharmacology* **2009,** *202*, 411–7. doi: 10.1007/s00213-008-1278-5
133. Dakhale, G.N.; Khanzode, S.D.; Saoji, A. Supplementation of vitamin C with atypical antipsychotics reduces oxidative stress and improves the outcome of schizophrenia. *Psychopharmacology* **2005**, *182*, 494–498. https://doi.org/10.1007/s00213-005-0117-1.
134. Hoffer, L.J. Vitamin therapy in schizophrenia. *Isr. J. Psychiatry Relat. Sci.* **2008**, *45*, 3–10.
135. Sandyk, R.; Kanofsky, J.D. Vitamin C in the Treatment of Schizophrenia. *Int. J. Neurosci.* **1993**, *68*, 67–71. https://doi.org/10.3109/00207459308994261
